# Txnip/PDK4‐Mediated Hyperdependence on Fatty‐Acid Oxidation Amplifies Susceptibility of Diabetic Myocardium to Ischemia–Reperfusion Damage

**DOI:** 10.1111/1753-0407.70277

**Published:** 2026-10-20

**Authors:** Zheng Zeng, Wei Shi, Yuxuan He, Yue Wang, Cong Pu, Jingtong Tan, Hai Guo, Haiping Ma

**Affiliations:** ^1^ Department of Anesthesiology The First Affiliated Hospital of Xinjiang Medical University Xinjiang China; ^2^ Catheterization Laboratory, Changji Prefecture People's Hospital, Changji Hui Autonomous Prefecture Xinjiang China

**Keywords:** diabetic myocardium, heterogeneity, I/RI, metabolic reprogramming, myocardial vulnerability

## Abstract

**Background:**

Undergoing perioperative ischemia‐reperfusion injury (I/RI), the prevalence of adverse cardiac events is high due to increased myocardial vulnerability in diabetic patients. However, the specific mechanisms of increased myocardial vulnerability in diabetes are still unknown.

**Objective:**

To investigate the specific mechanisms underlying increased myocardial vulnerability in diabetes and to explore potential myocardial protective strategies.

**Methods:**

In this study, we repeatedly verified through single‐cell sequencing, fluorescence in situ staining, metabonomics, and animal experiments.

**Results:**

Here we show for the first time that the cardiomyocyte subtype (C6 cardiomyocyte) is a cardiomyocyte characterized by myocardial contraction, and the reduction of its number and metabolic heterogeneity is the basis of myocardial ischemia‐reperfusion injury in diabetes. Among them, TXNIP/PDK4 mediated metabolic reprogramming is an important reason for myocardial vulnerability in diabetes, and through regulating TXNIP/PDK4, we optimize myocardial energy metabolism. It can significantly improve the vulnerability of diabetes myocardium, play a good role in myocardial protection, and has clinical transformation potential.

## Highlights

This study is the first to show that reduced C6 cardiomyocyte numbers and metabolic heterogeneity underlie increased myocardial vulnerability to ischemia–reperfusion injury in diabetes. TXNIP/PDK4‐mediated metabolic reprogramming is a key mechanism; regulating this pathway optimizes myocardial energy metabolism, significantly alleviating vulnerability, and conferring cardioprotection with clinical translation potential.

## Graphical abstract

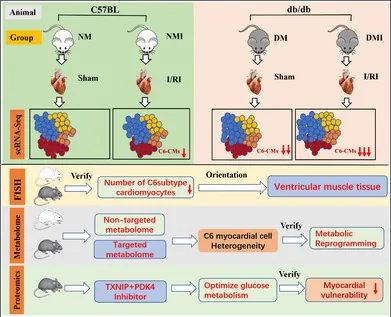

## 1 Introduction

Diabetes has become a serious social public problem endangering human health, especially its cardiovascular complications, which are the biggest challenge, accounting for 50%–80% of the deaths of diabetes patients [1, 2, 3]. The study found that the incidence of perioperative adverse cardiac events (myocardial ischemia, malignant arrhythmia, myocardial infarction, and heart failure) in diabetes patients was about five times higher than that in non‐diabetes patients [4]. The high incidence of perioperative adverse cardiac events in diabetes patients is mainly due to the increased vulnerability of diabetes myocardium to I/RI, which is one of the risk factors affecting and restricting the perioperative safety of such patients.

Currently, research on the increase of myocardial vulnerability in diabetes mainly focuses on exploring pathophysiological mechanisms, including energy metabolism disorder, oxidative stress, intracellular calcium overload, apoptosis, autophagy, signal pathway damage, and iron death [5, 6, 7]. These mechanisms present a complex process of multilevel, multifactorial, and interactive interactions between genes, molecules, cells, and tissues. Some studies have shown that myocardial energy metabolism disorder is the initial link and important mechanism of inducing myocardial I/RI in diabetes [8, 9]. Both animal and clinical experiments have confirmed that optimizing energy metabolism, particularly promoting glucose metabolism, can help alleviate I/RI and exert myocardial protective effects [10, 11]. However, the real reason and molecular mechanism of myocardial vulnerability increase caused by myocardial energy metabolism disorder in diabetes are still unclear.

Cellular metabolic reprogramming is closely related to the occurrence and progression of diseases and is currently one of the hotspots in life science research. Under physiological conditions, free fatty acid oxidation provides 60% to 80% of the total myocardial energy consumption, glucose oxidation provides 10% to 20%, and the rest is mainly provided by lactic acid and other sources. At the same time, the heart has considerable substrate metabolism flexibility [12]. The glucose metabolism of diabetes myocardium is inhibited, fatty acid metabolism is increased, and the effective utilization rate of sugar and fatty acid is significantly changed. This reprogramming of energy metabolism impairs myocardial function [13]. Research has shown that for a considerable period after myocardial ischemia/reperfusion, cardiac muscle cells mainly rely on glycolysis to obtain energy, and it has been found that increasing the concentration of glycolytic substrates in the blood circulation can alleviate cardiomyocytes damage and promote myocardial function recovery [14, 15]. However, the specific molecular mechanism of myocardial energy metabolism reprogramming leading to increased myocardial vulnerability in diabetes is still unclear.

This study first used single‐cell RNA sequencing (scRNA‐seq) technology to comprehensively understand the changes in the transcriptome of genes related to energy metabolism in diabetes myocardium I/RI, reveal the molecular mechanism between myocardial metabolic reprogramming and myocardial vulnerability, and then verified it at the tissue level through FISH and metabonomics technology. Finally, in animal experiments, we explored whether we could improve the vulnerability of diabetes myocardium by regulating myocardial I/RI energy metabolic reprogramming in diabetes myocardium and lay the foundation for clinical application transformation.

## 2 Results

### 2.1 The scRNA‐seq Results Showed the Changes in the Number and Functional Heterogeneity of Cardiomyocyte Subtypes Were the Material Basis for Increased Myocardial Vulnerability After Diabetic Myocardial I/RI

Cell heterogeneity has become a focus for the study of cardiac physiology and pathological mechanisms [16, 17, 18, 19, 20, 21]. To explore the heterogeneity of diabetic cardiomyocytes, using the 10× Genomics Chromium system, the heart tissues of the four groups of healthy C57BL/6J mice (NM), C57BL/6J I/RI mice (NMI), diabetic db/db mice (DM), and diabetic db/db I/RI mice (DMI) were subjected to scRNA‐seq using the cell nucleation technique. A total of 48 320 nuclei were extracted, of which 40 645 nuclei were finally retained after a quality control screening process and used to create a scRNA‐seq dataset. The scRNA‐seq results were subsequently analyzed (see Appendix S1 for details). Twenty‐six cell subgroups were identified in heart tissue. The cell types were annotated using the scRNA‐seq cell type annotation software Single R [22] and manually corrected with marker gene studies. The 26 clusters were annotated into 11 cell types: cardiomyocytes, endothelial cells, fiber cells, smooth muscle cells, pericytes, cardiac glial cells, epicardial cells, neural cells, glial cells, T cells, and B cells (Figure 1A).

**FIGURE 1:**
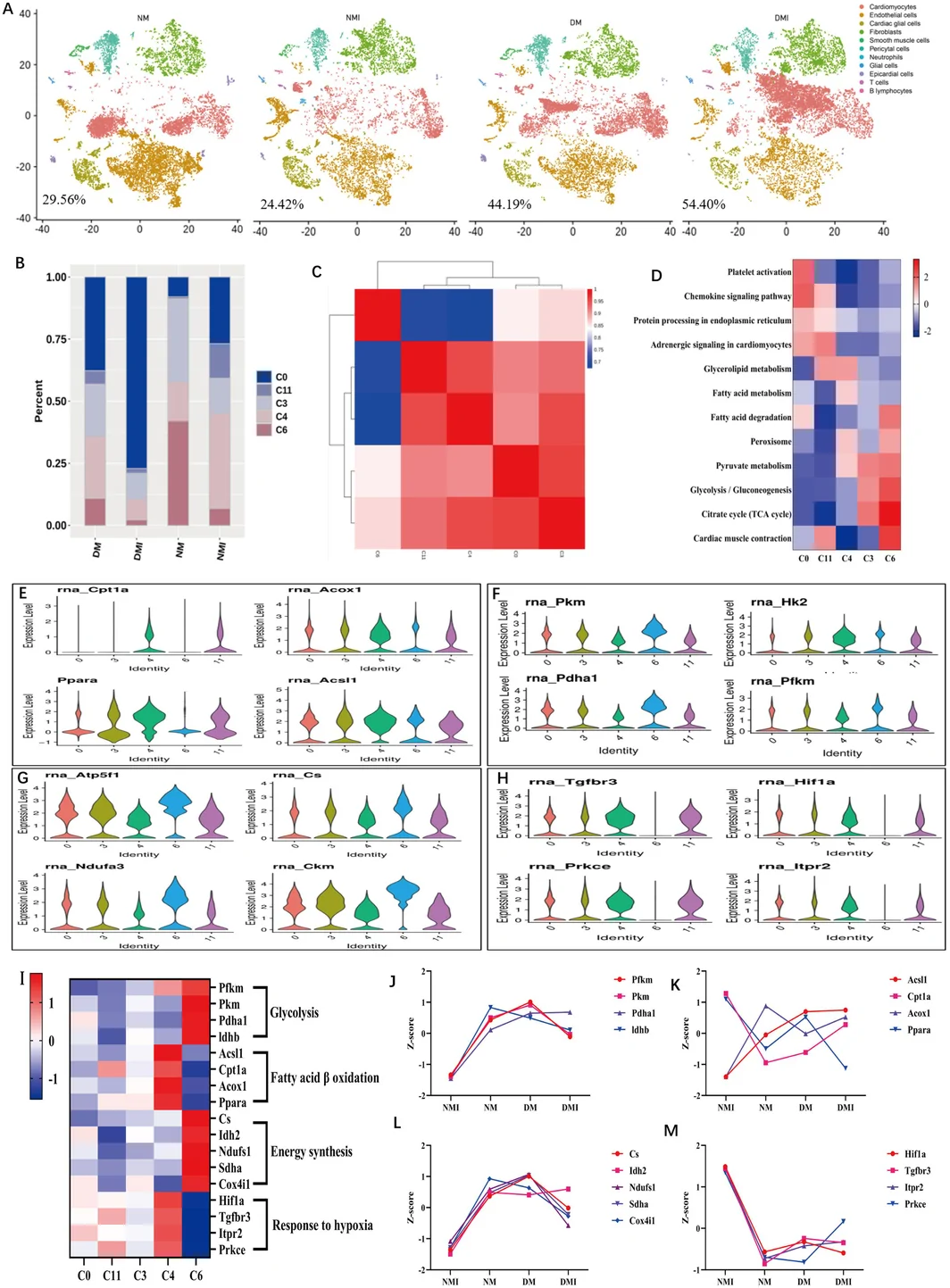
The number of C6‐CMs cells decreased after myocardial I/RI in diabetic patients. Enhanced fatty acid metabolism is the material basis of increased myocardial vulnerability. (A) t‐SNE plot shows 11 cell subgroups, NM: Healthy C57BL/6J mice group; NMI: Healthy C57BL/6J I/RI mice group; DM: Diabetic mice group; DMI: Diabetic db/db I/RI mice group; the red in the plots is the cardiomyocyte subgroups; the labeled numbers are the proportions of cardiomyocytes. (B) Histogram showing the proportions of the five cardiomyocyte subgroups under different conditions. (C) Correlation heat map of the five cell subtypes. (D) GSEA heatmap of the five cardiomyocyte subtypes. (E) Violin plot of genes related to fatty acid metabolism. (F) Violin plot of genes related to glucose metabolism. (G) Violin plot of genes related to energy synthesis. (H) Violin plot of genes related to hypoxia resistance. (I) Heatmap of gene transcription levels in the C6‐CMs. (J) Trends of key glucose metabolism genes in the C6‐CMs. (K) Trends of genes related to fatty acid ꞵ‐oxidation in the C6‐CMs. (L) Trends of key energy synthesis genes in the C6‐CMs. (M) Trends of hypoxia response genes in the four C6‐CMs.

Analysis of the proportion of different types of cells among the four groups revealed that cardiomyocytes (38.14%), endothelial cells (29.52%), and fibroblasts (16.36%) were the main types of cells that make up the heart tissue, accounting for 80% of the total number of cardiac cells. The difference in cardiomyocytes among the four groups was the greatest, with the NM group accounting for 29.56% of cardiomyocyte subsets, the NMI group accounting for 24.42%, the DM group accounting for 44.19%, and the DMI group accounting for 54.40% (Figure 1A,B). In terms of cardiomyocytes number, the NMI group had fewer cardiomyocytes than did the NM group due to cardiomyocytes apoptosis caused by I/RI, which is logical, and changes in the cardiomyocytes in the diabetes groups were inconsistent with conventional logic (specific reasons are provided in Appendix S2).

Cardiomyocytes were further subdivided into five subtypes according to marker genes, that is, the C0, C3, C4, C6, and C11 subtypes, based on marker genes (Figure 1B,C, the list of Marker genes is attached), with significant changes in the proportions of the C6‐cardiomyocyte subtype (C6‐CMs) among the four groups. The C6‐CMs accounted for the highest proportion of cells in the NM group (42.1%), with a significantly lower proportion in the NMI group (6.8%); the proportion in the DM group (10.8%) was significantly lower than that in the NM group, and the lower proportion of this subtype in the DMI group (2.2%) compared with that in the DM group was particularly notable. These results indicate that the C6‐CMs is the most predominant cardiomyocyte subtype in healthy myocardium, with significantly higher proportions than in diabetic myocardium, and that the number of C6‐CMs after I/RI significantly decreased, an effect due to cardiomyocyte apoptosis after I/RI. The analysis showed that the C6‐CMs were the most damaged cardiomyocyte type after I/RI, and the damage is more severe in diabetic myocardium.

To further analyze the functional characteristics of the five cardiomyocyte subtypes, the correlations and cross‐relationships of highly expressed differentially expressed genes (DEGs) among groups were analyzed, and gene set enrichment analysis (GSEA) was performed. The results showed that the C6‐CMs were significantly different from other cardiomyocyte subtypes and a relatively functionally independent cardiomyocyte type (Figure 1C). GSEA showed that functions of the C6‐CMs were mainly concentrated in pyruvate metabolism, the tricarboxylic acid (TCA) cycle, and myocardial contraction (Figure 1D) while pyruvate metabolism and tricarboxylic acid circulation are the necessary prerequisites to maintain the continuous contractile function of the myocardium. Based on these, we reasonably infer that the C6‐CMs are a cardiomyocyte type whose main function is myocardial contraction.

In normal cardiomyocytes, the fatty acid ꞵ‐oxidation pathway in mitochondria is the main mode of energy acquisition by the myocardium. Therefore, key fatty acid oxidation genes (Acsl1, Cpt1a, Acox1, and Ppara) were analyzed, and the results showed that the expression levels of key fatty acid metabolism genes in the C6‐CMs were significantly lower than those in other cardiomyocyte subtypes (Figure 1E). Glucose metabolism in normal cardiomyocytes is another important pathway for energy acquisition by the myocardium. A comparison of the expression levels of key glucose metabolism genes (Hk2, Pfk‐1, Pkm, and Pdha1) in the C6‐CMs with those in other cardiomyocyte subtypes showed that, except for glycolytic Hk2, the expression levels of other genes were significantly higher in C6‐CMs than in other cardiomyocyte subtypes (Figure 1F). These results indicate that the C6‐CMs preferentially use glucose as the main substrate for energy metabolism.

The TCA cycle and electron transport chain are important processes for energy synthesis; therefore, the transcription levels of key genes (Cs, Atp5f1, Ndufa3, and Ckm) were compared. The results showed that the transcription levels of key genes in the C6‐CMs were significantly higher than those in other cardiomyocyte subtypes (Figure 1G). The expression levels of hypoxia response‐related genes (Tgfbr3, Hif‐1α, Prkce, and Itpr2) were all low in the C6‐CMs (Figure 1H). These results indicate that the C6‐CMs have strong energy synthesis capabilities but relatively poor resistance to ischemia/hypoxia; this functional characteristic is consistent with the result that the C6‐CMs is a cardiomyocyte type mainly characterized by myocardial contraction, which again confirms that the C6‐CMs may be the most vulnerable cardiomyocyte type after I/RI.

To further explore the metabolic characteristics of the C6‐CMs, the trends of the key genes in different states (among the four groups) were analyzed. The results showed that among the key genes in the regulation of fatty acid metabolism (Figure 1I,K), except for Acox1, the expression of the remaining three was significantly lower in the NM group than in the DM group; Acsl1 and Acox1 expression was significantly lower in the NMI group than in the NM group and significantly higher in the DMI group than in the DM group. Ppara and Cpt1a, as key enzymes involved in the regulation of fatty acid metabolism, were significantly up‐regulated in healthy and diabetic myocardial tissues after I/RI. The results showed that the index of fatty acid metabolism decreased in healthy myocardium after I/RI, but increased in diabetic myocardium after I/RI.

The key genes involved in glucose metabolism were significantly downregulated in both the NMI and DMI groups, but the downregulation in the DMI group was significantly smaller than that in the NMI group, indicating a significant decrease in glucose metabolism ability in healthy myocardium after I/RI. However, after I/RI in diabetes, there was little change in glucose metabolism. Therefore, in summary, it is believed that the glucose and fatty acid metabolism abilities of healthy myocardium significantly decrease after I/RI. After myocardial I/RI in diabetes, glucose metabolism decreased, while fatty acid metabolism continued to increase. It indicates that the diabetes myocardium excessively relies on fatty acid as an energy substrate for energy supply after I/RI (Figure 1I,K).

Energy synthesis‐related genes were significantly downregulated in the NMI group and DMI group, but the downregulation in the DMI group was less than that in the NMI group (Figure 1I,L). The hypoxia response genes Hif‐1α, Tgfbr3, Prkce, and Itpr2 were all significantly upregulated in the NMI group; in the DMI group, only Prkce was upregulated, and the other genes were all downregulated. These results show that the ability of healthy myocardium to withstand ischemia/hypoxia is significantly higher than that of diabetic myocardium, which may be one of the reasons for the increased vulnerability of diabetic myocardium (Figure 1I,M).

To screen the genes that cause the greatest increase in myocardial vulnerability after diabetic myocardial I/RI, the principle of “considering both pathological changes in diabetes and the damage of I/RI” was followed in the screening of target genes for further analysis. Txnip is the representative gene mainly reflecting the influence of pathological changes in diabetes, and Pdk4 is the representative gene mainly reflecting the influence of I/RI. Txnip and Pdk4 were identified by analyzing the DSEs of the C6‐CMs (see Appendix S3 for details).

In summary, the scRNA‐seq results suggested that the reduction in the C6‐CMs and the heterogeneity in energy metabolism in the diabetic myocardium are the material bases of increased myocardial vulnerability; the metabolic reprogramming of diabetic myocardial tissues caused by Txnip/Pdk4 is a key link in increased myocardial vulnerability after I/RI. The next step is to validate the spatial localization and metabolomics of C6 cardiomyocyte subtypes in different parts of the heart.

### 2.2 Fluorescence In Situ Hybridization (FISH) Was Used to Localize C6 Cardiomyocyte Subsets Mainly in Ventricular Muscle Tissue and Verify the Difference in Cell Number Between Healthy and Diabetes Myocardium

Although scRNA‐seq has revolutionized the ability to define different cellular states and diversity [23, 24], due to the special shape and structure of cardiomyocytes, it is technically difficult to sort adult cardiomyocytes. Fluorescence in situ hybridization (FISH) is a reliable technique for cell localization and number analyses [25]. The combination of FISH and scRNA seq gene information can achieve the identification and screening of cardiomyocyte subtypes. The study uses FISH to achieve spatial localization of C6‐CMs and analyze differences in cell numbers.

To identify the C6‐CMs in myocardial tissue, using scRNA‐seq data, Ttn, Myl3, and Xirp2 were screened as marker genes for the spatial positioning of the C6‐CMs (see Appendix S4 for details). According to the scRNA‐seq functional analysis of cardiomyocyte subtypes, the main function of the C6‐CMs is contraction. Therefore, it is speculated that the C6‐CMs may be the most important cardiomyocyte subtype within healthy ventricular muscle tissue. Based on this, q‐PCR detection was performed on the heart tissue of healthy C57BL/6J mice to verify the differential expression of Ttn, Myl3, and Xirp2 in the atria and ventricles of the heart, thereby confirming the reliability of the above markers as representative genes for C6‐CMs. The q‐PCR results showed (Figure 2I–K) that the expression levels of Ttn, Myl3, and Xirp2 in ventricular tissue were significantly higher than those in atrial tissue. Based on this, we determined that Ttn, Myl3, and Xirp2 could be representative marker genes of the C6‐CMs. FISH was performed to study the spatial positioning of the C6‐CMs (see Appendix S5 for details).

**FIGURE 2:**
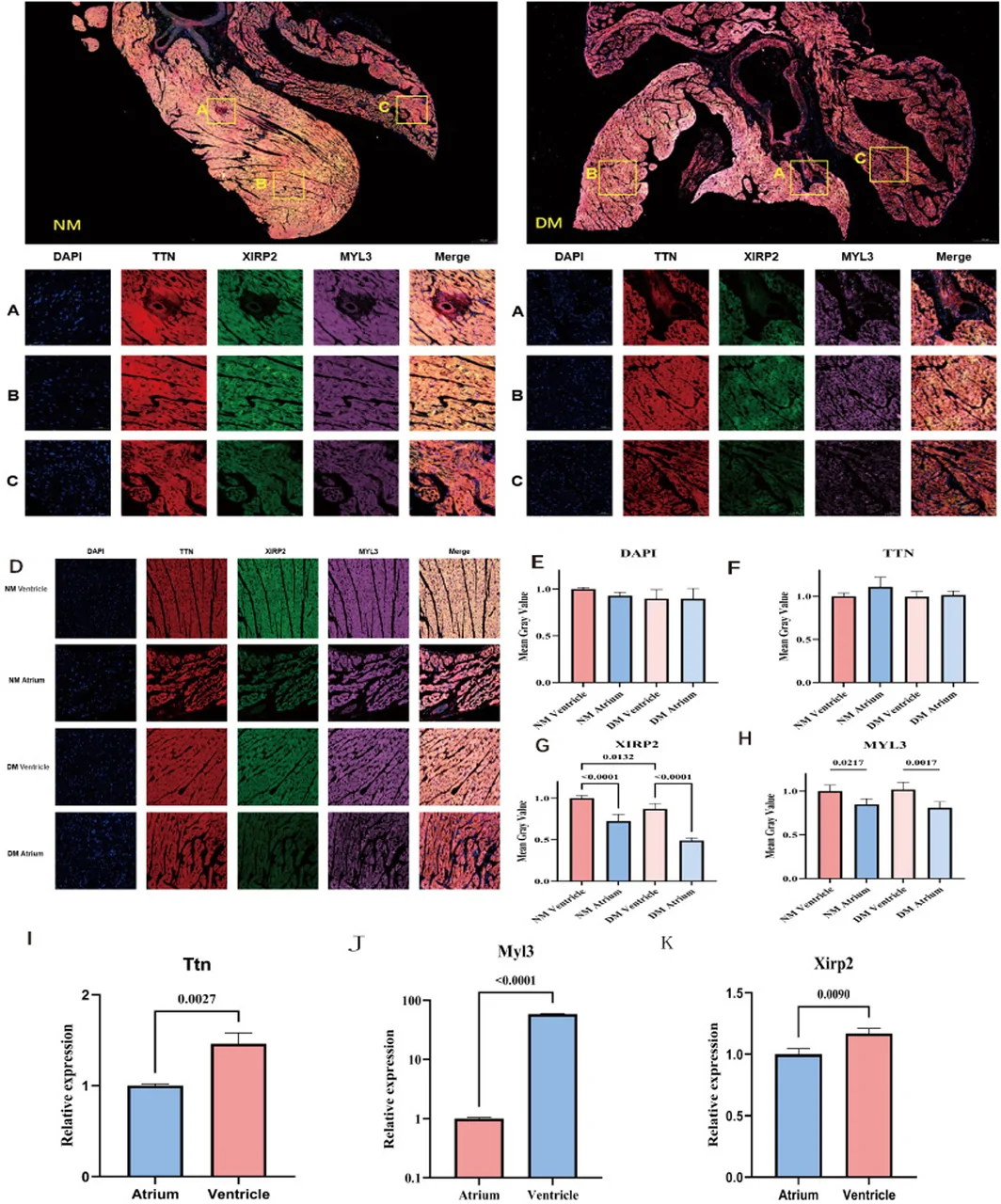
C6‐CMs were mainly localized in ventricular myocytes and varied in number between groups. *n* = 3 in each group; original magnification ×7.5; NM: Heart tissue of a C57BL/6J mice; DM: Heart tissue of a db/db mouse; (A) blood vessels; (B) ventricular tissue; (C) atrial tissue; nuclei stained with 4′,6‐diamidino‐2‐phenylindole (DAPI) are blue under excitation with ultraviolet (UV) light; positive expression is fluorescein fluorescence. Xirp2 is green, Ttn is red, and Myl3 is purple; (D–H) FISH images of atrial and ventricular tissue from the NM group and DM group; original magnification ×63; (I–K). Comparative q‐PCR analysis of Ttn, Myl3, and Xirp2 in the different groups (*p < 0.05, **p < 0.01, ***p < 0.001).

To achieve spatial localization of C6 cardiomyocyte subtypes, it is first necessary to distinguish between cardiomyocytes and noncardiomyocytes in cardiac tissue. Using FISH multi‐staining technology, heart tissues from NM and DM mice were fluorescently stained, and the atrium, ventricular muscle, and vascular tissues were all visible. The vascular tissue (Figure 2A), ventricular muscle (Figure 2B), and atrial muscle (Figure 2C) were analyzed under 7.5 times magnification, and the results showed that the myocardial tissue had significantly more positive cells than did vascular tissue in the two groups, indicating that cardiomyocytes were successfully identified in heart tissue. Therefore, Ttn, Myl3, and Xirp2 can be used as marker genes to identify cardiomyocytes by FISH.

To compare the differences in the number of C6‐CMs in atrial and ventricular tissues, FISH was performed for the NM group (C57BL/6J mice, *n* = 3) and DM group (db/db mice, *n* = 3). The results showed (Figure 2D–H) that as a marker for the identification of cardiomyocytes, Ttn was significantly highly expressed in atrial and ventricular myocardial tissues, with no statistically significant difference in expression between the groups; as marker genes for the C6‐CMs, Xirp2, and Myl3 expression levels were significantly higher in the ventricle than in the atrial tissue in both the NM and DM groups, with statistically significant differences (*p* < 0.05). A comparison of the number of positive cells in myocardial tissue showed that the number of positive cells was significantly higher in ventricular tissue than in atrial tissue in the two groups. The results showed that Ttn, Myl3, and Xirp2, as marker genes for myocardial tissues, could identify cardiomyocyte types; Myl3 and Xirp2 could serve as marker genes for differentiating the C6‐CMs from other myocardial subtypes; and the C6‐CMs were mainly concentrated in ventricular tissue.

To further validate the difference in the C6‐CMs among ventricular myocytes between the NM group and the DM group, fluorescence quantitative analysis of Ttn, Myl3, and Xirp2 in ventricular was performed (Figure 2D–H). The results showed that for Ttn, as a universal marker of cardiomyocytes, there was no statistically significant intra‐ and inter‐group difference; Myl3 was significantly higher in the ventricle than in the atrium for the NM group and the DM group, with statistical significance (*p* < 0.001), but there was no significant difference between the NM group and the DM group; and there was a statistically significant difference in Xirp2 between the ventricle and atrium for the NM group and the DM group (*p* < 0.01), and Xirp2 expression was significantly higher in the NM group than in the DM group (*p* < 0.05). It should be noted that the FISH signals for Ttn, Myl3, and Xirp2 were broadly detectable across cardiomyocytes, consistent with their roles as core structural/contractile genes. However, quantitative comparisons of signal intensity and positive cell counts revealed significant regional and intergroup differences, supporting their utility as quantitative markers for C6‐CM abundance rather than as binary‐specific labels. The results showed that Myl3 and Xirp2 could be used to distinguish atrial and ventricular myocytes, and Xirp2 was significantly overexpressed in NM myocardium, suggesting that C6‐CMs were significantly higher in healthy ventricular myocytes than in diabetic myocardium. FISH once again confirmed the difference in the number of C6‐CMs between the NM and DM groups, which is consistent with scRNA‐seq analysis.

In this study, FISH was used to find that the spatial localization of C6‐CMs in heart tissue was mainly in ventricular tissue, and the number of C6‐CMs in healthy myocardium was higher than that in diabetic myocardium, which verified the difference in the number of C6‐CMs cells in early scRNA‐seq. The next step is to verify the relationship between the heterogeneity of energy metabolism of C6 cardiomyocyte subtypes and the myocardial I/RI vulnerability in diabetes.

### 2.3 Metabolomics Was Used to Validate the Differences in the Selected Metabolic Substrates in Diabetes Ventricular Muscle Tissue

Changes in energy substrates utilized by the heart may be the basis of the significant changes in the structural remodeling and function of myocardial tissues [26, 27]. Metabolomics research serves as a reliable means of verifying gene transcriptomics results. In this study, non‐targeted metabolomics was first used to detect and analyze ventricular muscle tissue from 4 groups of 20 samples (Figure 3A). A total of 815 metabolites were detected, clustered into 14 categories and 6 major groups. Principal component analysis (PCA) indicated that the four clusters were well separated. Among them, 18 differential metabolites of fatty acids and glucose were screened (Figure 3B,C), with 13 upregulated and 5 downregulated. Comparison of glucose and fatty acid differential metabolites in the NM and DM groups revealed four metabolic differentials: propionylcarnitine, sengenoside, (R)‐‐3hydroxy‐tetradecanoic acid and adrenoic acid; among them, sengenoside from the glucose metabolite as well as the (R)‐‐3hydroxy‐tetradecanoic acid and adrenoic acid from the fatty acid metabolites were significantly higher expressed in the NM group, and the fatty acid metabolites (R)‐‐3hydroxy‐tetradecanoic acid and adrenoic acid were significantly highly expressed in the NM group, and propionylcarnitine was significantly highly expressed in the DM group; comparing the NMI group with the DMI group, except for the organic acid 3‐hydroxyglutaric acid, which was highly expressed in the NMI, five lipid metabolites (suberic acid, dodecanedioic acid, caproic acid, 12‐hydroxystearic acid and octadecenoic acid) were highly expressed in the DMI group. It shows that the energy of the myocardium is mainly supplied by fatty acid metabolism and a small part of glucose is supplied by healthy myocardium, but the glucose metabolism of diabetes myocardium is significantly reduced, and diabetes myocardium is more dependent on fatty acid metabolism to obtain energy after I/RI.

**FIGURE 3:**
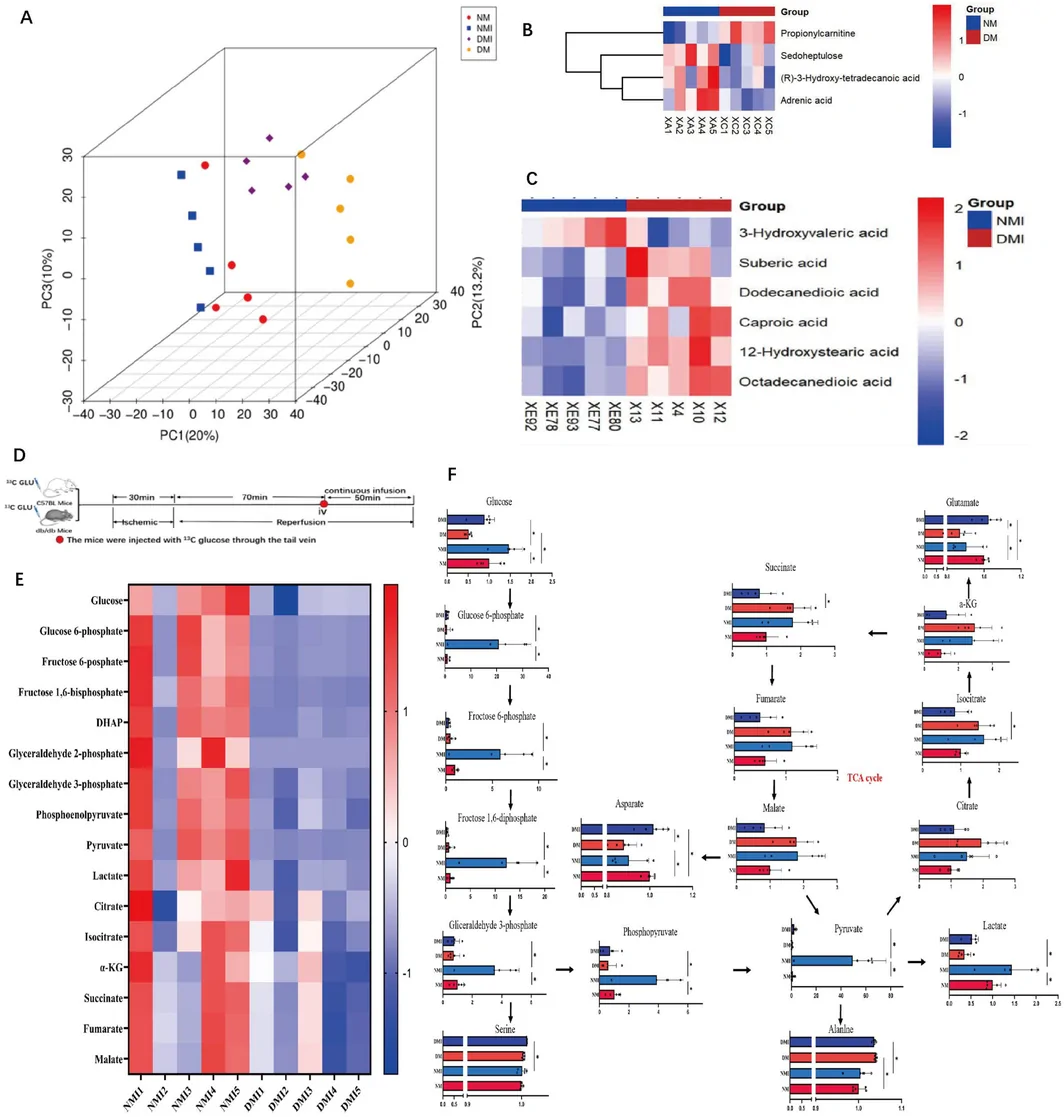
Diabetic myocardial I/RI is mainly dependent on fatty acid metabolism. *n* = 5 in each group. (A) Scatter plot (PCA) of the 3D model. (B) Cluster heatmap of differential metabolites of glucose and fatty acids between in NM and DM. (C) Cluster heatmap of glucose and fatty acids between in NMI and DMI. (D) Experimental procedures. (E) Cluster heatmap of differential metabolites between in NMI and DMI. (F) Comparative analysis of ^13^C glucose‐labeled metabolites in the tricarboxylic acid cycle between four groups.

Stable isotope labeling is an important tool for studying metabolic flux. ^13^C isotope‐labeled metabolites can be used to study the contribution ratio of different substrates to the TCA cycle under different conditions and in different tissues and to explore possible changes in substrate selection under different pathological and physiological conditions [28, 29]. To further clarify the difference in glucose utilization by myocardial tissue under different conditions, isotope‐labeled ^13^C6‐glucose was injected into animals in each group (*n* = 5), and the metabolic flux of glycolytic and TCA cycle intermediates in cardiac tissue was assessed using targeted metabolomics (Figure 3D–F). In ^13^C6‐glucose flux analysis, the measured levels of ^13^C6‐labeled glycolytic and TCA cycle intermediates in healthy myocardial were significantly higher than those in diabetic myocardium, and the glucose metabolites in diabetic myocardial tissues after I/RI were significantly lower than those in the healthy group (*p* < 0.05). The results showed that there were significant differences in glucose utilization between healthy and diabetic myocardial tissues after I/RI. Increased glucose metabolism leads to the inhibition of fatty acid metabolism through the Randle cycle (a competitive mechanism of fatty acid and glucose). Therefore, the study suggests a higher dependence on fatty acids for energy metabolism in diabetic myocardial tissues (detailed analysis can be found in Appendix S6).

### 2.4 Regulation of Diabetic Myocardial I/RI Energy Metabolism Through TXNIP/PDK4 to Attenuate Myocardial Injury

The scRNA‐seq results showed that the Txnip/Pdk4 genes in the C6‐CMs were significantly highly in diabetic myocardium after I/RI and that the expression of these genes is an important factor causing the reprogramming of myocardial energy metabolism. The overexpression of TXNIP caused by the increase of glucose levels in the serum of mice is a typical manifestation of diabetes and insulin resistance and will lead to cytotoxicity. This process is relatively long. In this study, verapamil and dichloroacetic acid (DCA) were chosen to inhibit TXNIP and PDK4, respectively. Six‐week‐old db/db mice were administered verapamil 3 weeks before the experiment and then grouped. The results showed that TXNIP inhibitor verapamil feeding had a significant effect on body weight and glucose in db/db mice, which may have led to a reduction in the extent of diabetic myocardial lesions (Figure 4, Table 3 in Appendix S6).

**FIGURE 4:**
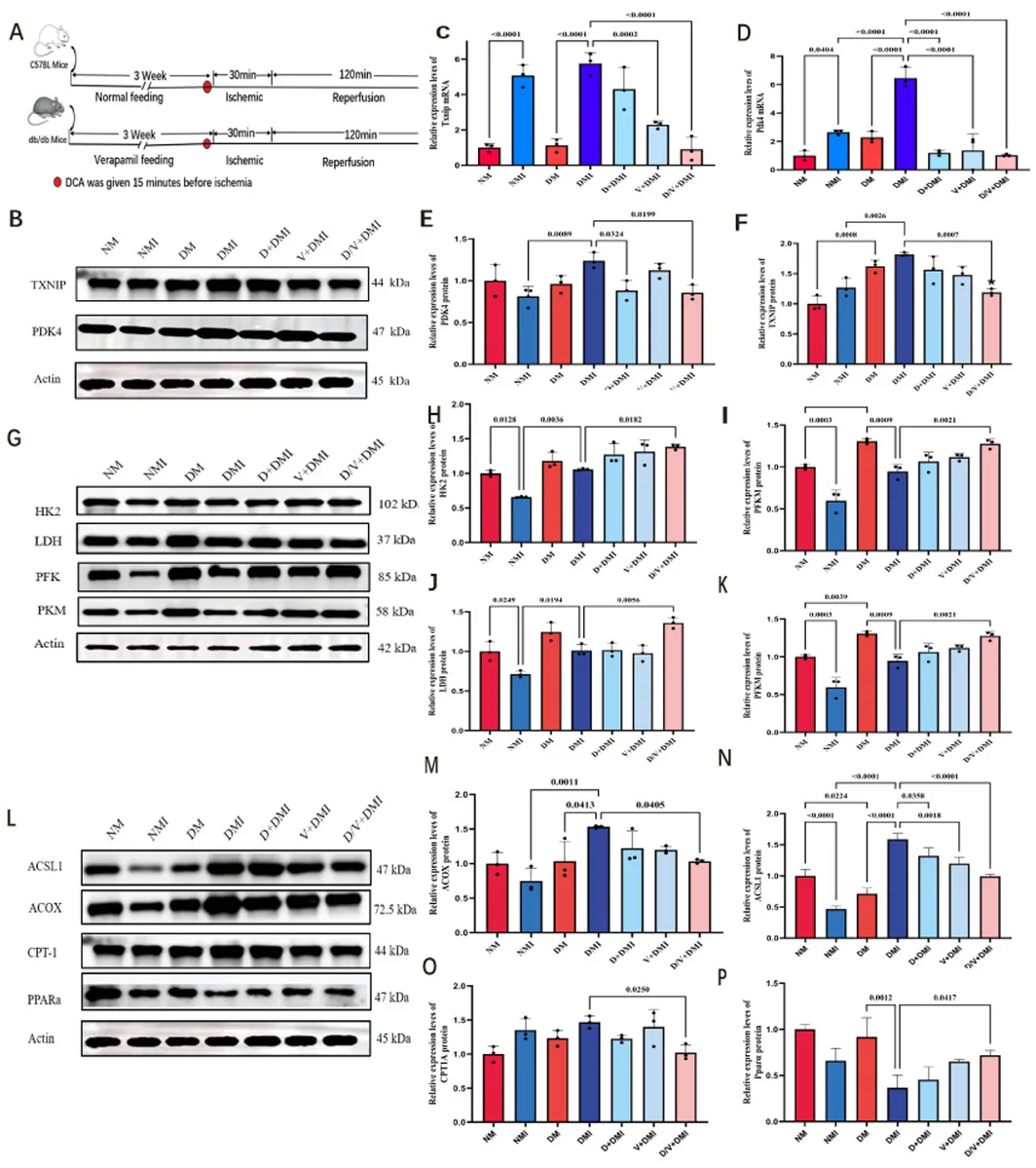
After the intervention of TXNIP/PDK4 inhibitors, the glucose metabolic capacity of healthy and diabetic myocardial I/RI increased, and the fatty acid metabolism of diabetic myocardial decreased, alleviating the myocardial injury after I/RI. *n* = 3 in each group. (A) Experimental process. (B, C) Q‐PCR analysis of TXNIP and PDK4 mRNA expression at different groups. (D–F) Western blot analysis of TXNIP and PDK4 protein expression at different groups. (G) Western blot analysis of proteins associated with glucose metabolism at different groups. (H–K) The histogram shows the levels of HK, LDH, PFK, and PKM proteins in different groups. (L–P) Western blot analysis of proteins associated with fatty acid metabolism at different groups. D + DMI: Dichloroacetic acid intervention in db/db ischemia–reperfusion group mice; V + DMI: Verapamil intervention in db/db ischemia–reperfusion group mice; D/V + DMI: Dichloroacetic acid and Verapamil combined intervention in db/db ischemia–reperfusion group mice. ^: vs. NM, &: vs. NMI, *: vs. DMI, #: vs. DM.

To assess the effect of TXNIP/PDK4 inhibitors on glucose metabolism after myocardial I/RI. The expression of TXNIP and PDK4 mRNA was detected by q‐PCR, and the protein expression levels of TXNIP and PDK4 were detected by western blot (WB) (Figure 4B–F). The results showed that TXNIP and PDK4 mRNA were increased significantly in both the NMI and DMI groups than in the NM and DM groups respectively, especially in the DMI group. After administration of TXNIP/PDK4 inhibitors verapamil and acetoacetic acid (DCA), the expression levels of TXNIP and PDK4 mRNA were significantly lower than DMI groups, with statistically significant differences (*p* < 0.05). Similarly, the protein expression level of TXNIP/PDK4 was significantly lower than DMI group after administration of TXNIP/PDK4 inhibitors verapamil and DCA (*p* < 0.05), which was consistent with the results of scRNA‐seq.

To evaluate the effect of TXNIP/PDK4 intervention on myocardial glucose metabolism in diabetes, WB was used to detect the changing trend of key enzymes of glucose metabolism in each group (*n* = 6 in each group) (Figure 4G–K): pyruvate dehydrogenase (PDH), hexokinase 2 (HK2), phosphofructose kinase 1 (PFK1), pyruvate kinase M2 (PKM2), and lactate dehydrogenase A (LDHA). The results showed that, except LDHA in the DM group was higher than that in the NM group (*p* < 0.05), the rest of PDH, HK2, PFK1, and PKM2 in the DM group was slightly higher than that in the NM group, but there was no statistical difference (*p* > 0.05). The four key glucose enzymes mentioned above decreased after I/RI in healthy and diabetes myocardium, and there was no statistical difference (*p* > 0.05). The levels of PDH, HK2, PFK1, PKM2, and LDHA in the V + DMI group, D + DMI group, and D/V + DMI group were all increased compared to the NMI, DM, and DMI groups (*p* < 0.05). Among them, the increase in NMI was the largest, and the increase was particularly significant in the D/V + DMI group (*p* < 0.01). The results showed that the glucose metabolism ability of diabetes myocardium was significantly lower than that of healthy myocardium. The glucose metabolism ability of healthy myocardium decreased significantly after I/RI, while the glucose metabolism declined slightly after I/RI of diabetes myocardium. The glucose metabolism ability of healthy and diabetes myocardium increased after the intervention of TXNIP/PDK4 inhibitors.

After TXNIP/PDK4 inhibitor treatment, the function of fatty acid metabolism after myocardial I/RI was explored in animal experiments (*n* = 6 in each group). The following key enzymes of fatty acid metabolism were detected using WB (Figure 4L–P): acyl‐CoA oxidase 1 (ACOX1), long‐chain acyl‐coenzyme A synthetase 1 (ACSL‐1), carnitine palmitoyltransferase‐1 (CPT‐1) and peroxisome proliferator‐activated receptor a (PPARa). The results showed that ACSL‐1 and PPARa, as two key enzymes involved in fatty acid metabolism, were lower in the NMI group than in the NM group, while DMI was higher in the DM group. The increase in DMI compared to DM and NMI groups was statistically significant (*p* < 0.05); The protein CPT‐1, which is responsible for transporting fatty acids to mitochondria, increased after I/RI, and was significantly higher in the DMI group than in the DM group, with a statistically significant difference (*p* < 0.05); The PPARa protein, which regulates fatty acid metabolism, decreased after I/RI and showed a statistically significant increase compared to the DMI group after simultaneous administration of both inhibitors (*p* < 0.05); The expression levels of CPT‐1, ACSL‐1, and ACOX1 were significantly lower in the DMI group after administration of both intervention agents (*p* < 0.05). The results showed that the fatty acid metabolism of diabetes myocardium was further enhanced after I/RI, and after the intervention of TXNIP/PDK4 inhibitor, the fatty acid metabolism of diabetes myocardium could be significantly reduced.

### 2.5 Inhibition of TXNIP/PDK4 Preserves Cardiac Pump Function, Attenuates Apoptosis and Limits Myocardial Infarct Size After Diabetic I/RI

Pumping blood is the core function of the heart. Previous studies have found that metabolic reprogramming of diabetic myocardium after I/RI results in impaired heart function. In this study, cardiac ultrasound was used to evaluate the effect of TXNIP/PDK4 inhibitors on the ability of myocardial tissues to pump blood after I/RI. The results (Figure 5A–F) showed that heart rate (HR), stroke volume (SV), and cardiac output (CO) were not statistically significantly different among the groups for both healthy and diabetic myocardium after I/RI (*p* > 0.05); ejection fraction (EF) and left ventricular fractional shortening (FS) were slightly lower in the DM group than in the NM group, and those in the NMI and DMI groups were significantly lower than those in the NM and DM groups (*p* < 0.05); and EF and FS were significantly higher in the V + DMI group, the D + DMI group, and the D/V + DMI group than in the DMI group (*p* < 0.05) but not significantly different from those in the DM group (Table 4 in Appendix S6). The results indicated that vulnerability was significantly increased and cardiac function was significantly impaired after diabetic myocardial I/RI and that myocardial function improved after TXNIP/PDK4 treatment, with significant improvement after the combined use of TXNIP/PDK4 inhibitors.

**FIGURE 5:**
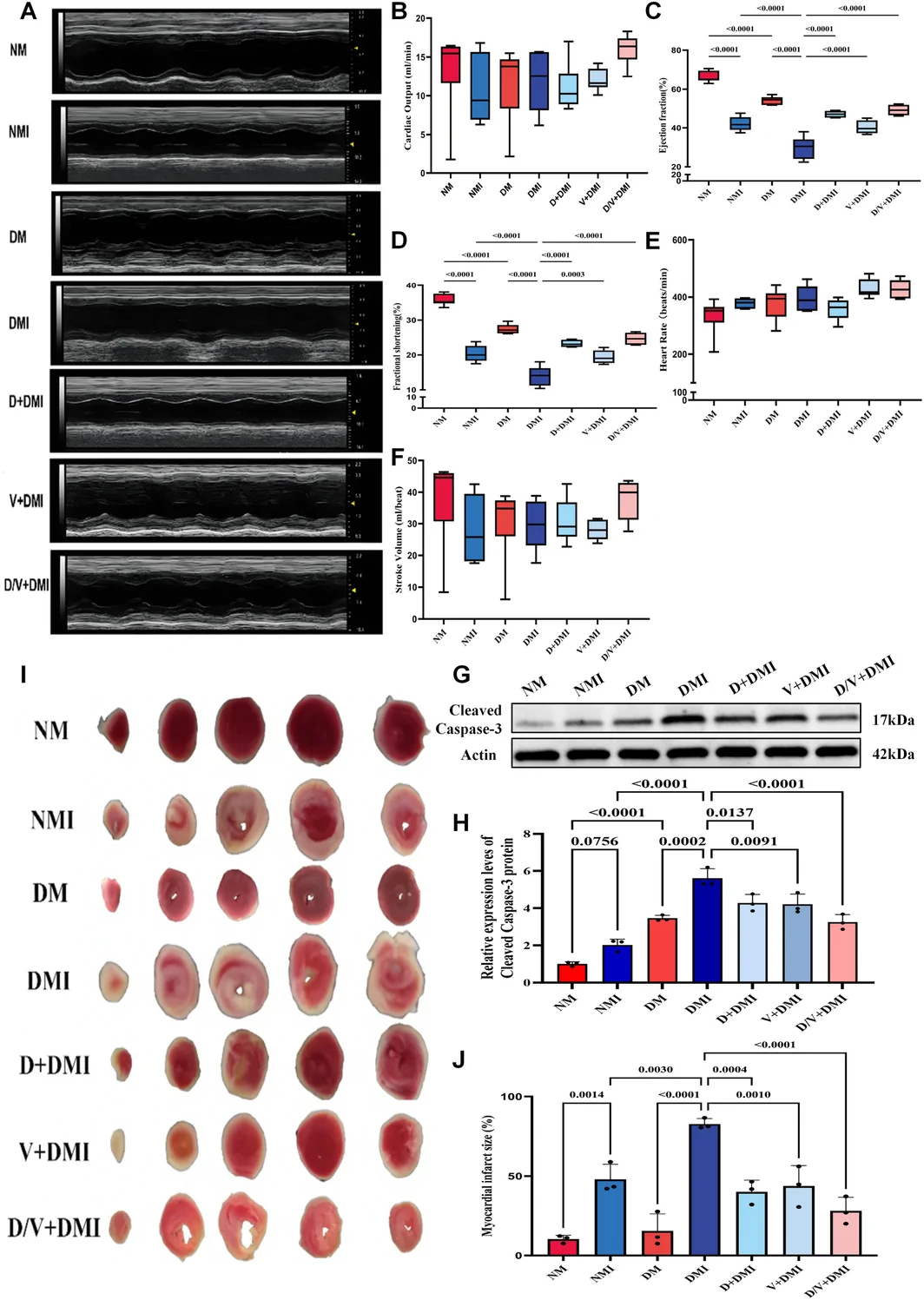
After TXNIP/PDK4 inhibitor intervention, diabetic myocardial I/RI showed enhanced cardiac function, decreased infarct area, and reduced apoptosis. *n* = 6 in each group. (A) Echocardiography in each group. (B–F) The box diagram shows the indicators of hypercardiography in each group. HR: Heart rate, SV: Cardiac volume, CO: Output per minute, EF: Ejection fraction, FS: Left ventricular brachialis shortening rate. (G) Cleaved caspase‐3 western blot bands of each group. (H) The histogram shows the levels of cleaved caspase‐3 proteins in different groups. (I) Representative TTC‐stained myocardial sections from each group (white areas indicate infarcted tissue, red areas indicate viable tissue). (J) Quantitative analysis of myocardial infarct size.

In this study, Western blot was employed to detect Cleaved Caspase‐3 protein expression in order to evaluate the effect of TXNIP/PDK4 inhibitors on apoptosis following I/RI in diabetic myocardium. The results (Figure 5G,H) showed that Cleaved Caspase‐3 expression was significantly higher in the DM group than in the NM group (*p* < 0.05). After I/RI, the increase in Cleaved Caspase‐3 expression in the DMI group was significantly greater than that in the NMI group (*p* < 0.05). Treatment with either D + DMI or V + DMI alone significantly reduced Cleaved Caspase‐3 expression compared with the untreated DMI group (*p* < 0.05), and the combined regimen (D/V + DMI) further decreased the expression to a level significantly lower than that achieved by either monotherapy (*p* < 0.05). These findings indicate that baseline apoptosis is elevated in the diabetic state and is markedly exacerbated by I/RI; moreover, TXNIP/PDK4 inhibition—whether as monotherapy or in combination—significantly suppresses apoptosis, with the combined treatment exerting a more pronounced synergistic antiapoptotic effect.

TTC staining is the most common technique for assessing myocardial infarction size. TTC staining was used again to assess the effects of I/RI myocardial infarction in mice after TXNIP/PDK4 inhibitor intervention. The results showed (Figure 5I,J) that the infarct size in the DMI group was significantly larger than that in the NMI group (*p* < 0.05) and that the infarct size in the V + DMI group, D + DMI group, and D/V + DMI group was significantly lower than that in the DMI group (*p* < 0.05), with the reduction in infarct size in the D/V + DMI group being significant (*p* < 0.01). These results show that the vulnerability of healthy myocardial tissues significantly increases after I/RI in diabetes and that the myocardial infarct size and vulnerability significantly improve after TXNIP/PDK4 inhibitor treatment. The combined experimental effect of the two is even more.

## 3 Discussion

Cardiac I/RI is an important cause of perioperative adverse cardiac events during cardiac and noncardiac surgery [5]. Studies have shown that in diabetic patients, perioperative adverse cardiac events after I/RI are significantly higher than those in nondiabetic patients, mainly due to the increased vulnerability of myocardial tissues in diabetes [30, 31]. The relative imbalance between glucose oxidation and fatty acid oxidation during myocardial ischemia–reperfusion injury may be an important factor in the occurrence of reperfusion‐related myocardial dysfunction [13]. However, the mechanism underlying increased myocardial vulnerability in diabetes is currently unclear. This study is the first time to use scRNA‐seq to study the gene transcriptome of heart tissue with I/RI in healthy myocardium and diabetes myocardium. The results show that the heterogeneity of C6‐CMs in terms of the number of cells, energy metabolism, and anti‐hypoxia damage is the material basis leading to the increase of myocardial vulnerability in diabetes. FISH verified that the C6‐CMs were predominantly localized in ventricular myocardial tissue and that the number of C6‐CMs was much higher in healthy myocardium than in diabetic myocardium. These results suggest that the C6‐CMs, as the cardiomyocyte subtype whose primary function is myocardial contraction, is the cell type most susceptible to I/RI. Targeted and untargeted metabolomics indicated that after I/RI, the healthy myocardium preferentially utilizes glucose as a metabolic substrate, the diabetic myocardium overly relies on fatty acids for energy metabolism, and myocardial function was damaged, indicating that the metabolic reprogramming of myocardial tissues in diabetes is an important mechanism leading to increased myocardial vulnerability after I/RI. In animal studies, energy metabolism in diabetic myocardium was optimized by the TXNIP and PDK4 treatment, which significantly increased the ability to resist I/RI.

Traditionally, the myocardium has four physiological characteristics: contractility, rhythmicity, conduction, and excitability. In this study, scRNA‐seq was performed on 40 645 cells of the whole heart, and the results showed that cardiomyocytes were the main cell type constituting heart tissue (accounting for 38% on average in the four groups and 29.6% in healthy heart tissue), a finding that is consistent with the results of the study by Wolfien et al. [32]. Cardiomyocytes can be divided into different subtypes. Genetic analyses have shown that each subtype has significant functional heterogeneity. Studies have shown that the myocardial function of various cardiomyocyte subtypes is not balanced and consistent and that different subtypes have their unique functional characteristics, which is different from the traditional view and an important finding of this study. The C6‐CMs is the main cardiomyocyte type in ventricular tissue, and the heterogeneity in functional and metabolic characteristics of the C6 cardiomyocyte subtype is an important basis for vulnerability in diabetic myocardial tissues after I/RI. The results of this study diverge from the traditional understanding of the function of cardiomyocytes and provide new ideas for the study of the mechanisms of heart diseases.

There are significant genetic and functional heterogeneities among different tissues and cells, contributing to overall organ development and normal physiological function and plasticity [33]. Research on cell heterogeneity has been critical in elucidating the role of cells in maintaining the integrity of organ functions under different environments and pathophysiological states [32]. In this study, the C6‐CMs preferentially utilize glucose as a substrate for energy metabolism, its energy synthesis capability far exceeds that of other cardiomyocyte types, and its main function is myocardial contraction; therefore, the C6‐CMs are considered the main component of healthy ventricular muscle. However, the expression of hypoxia resistance‐related genes is significantly low, and cardiomyocytes tend to undergo apoptosis after I/RI, thus impairing myocardial pump function. In this study, scRNA‐seq data were analyzed to provide valuable information for unraveling the mechanism underlying the increased vulnerability of myocardial tissues in diabetes.

In previous scRNA‐seq studies of myocardial tissues, independent scRNA‐seq analyses were performed on ventricular and atrial myocytes [34, 35, 36]. In studies on heterogeneity with the assumption that the locations of cardiomyocytes have been clarified, some important information is often ignored. In this study, a mixture of whole heart tissue samples was subjected to scRNA‐seq, and then, the cardiomyocyte subtypes were reconfirmed based on genetic features. The C6‐CMs identified in the study are considered to be the main cardiomyocyte type constituting the ventricular myocardium, but they are not the only cardiomyocyte type in the ventricular myocardium; there may be other cardiomyocyte subtypes involved. The C6‐CMs may be present elsewhere in the heart with different proportions, which explains that not only can the ventricular muscle pump blood but also have other physiological functions. This study made an important innovative contribution to the understanding of the composition of cardiomyocytes in different anatomical locations of heart tissues and found that the C6‐CMs are characterized by poor resistance to hypoxic injury; therefore, they are the cell type most susceptible to I/RI and need intensive protection. This study found that the number of C6‐CMs in diabetic myocardial tissues was very low and that the increase in apoptosis after I/RI led to impaired myocardial pumping function, which is considered an important cause of diabetic myocardial vulnerability.

FISH combines molecular biology, histochemistry, and microscopy [37, 38]. Increasingly more fluorescence imaging techniques and fluorescent dyes have provided more options for detecting and localizing cells in different organisms [39]. Combined with the scRNA‐seq data, FISH was used to visualize Ttn, Myl3, and Xirp2 in cardiomyocytes, allowing the differentiation and spatial localization of C6‐CMs in ventricular tissue, revealing more abundant C6‐CMs in healthy myocardium than in diabetic myocardium, and validating the transcriptomics results. The high expression levels of Ttn, Myl3, and Xirp2 in myocardial tissue have been reported in previous studies [40]. This study found that Myl3 and Xirp2 could be used as marker genes for C6‐CMs and that the proteins encoded by these two genes are both involved in myocardial contraction, further echoing the inference that the main function of the C6‐CMs is contraction. Previous studies have found that changes in the Myl3 and Xirp2 genes could cause myocardial diseases, which is consistent with the conclusions of this study [41, 42, 43].

Changes in energy substrate utilization in the heart may be the basis of significant changes in the structure and function of the remodeled heart [26, 27]. scRNA‐seq showed that ventricular tissue preferentially utilizes glucose as a metabolic substrate and that diabetic myocardium after I/RI over‐relies on fatty acid metabolism for energy. Fatty acid metabolism disorders are extremely harmful to the heart in diabetic patients and often cause cardiac dysfunction. Excessive fatty acid uptake and lipotoxicity can lead to myocardial remodeling. In addition, increased fatty acid oxidation can lead to an increased rate of oxygen consumption, causing the excessive production of reactive oxygen species, oxidative stress in the endoplasmic reticulum, and mitochondrial uncoupling [44].

To analyze the utilization of energy substrates in the heart, nontargeted metabolomics and ^13^C isotope‐labeled glucose‐targeted metabolomics were performed. The results showed that healthy and mature cardiomyocytes mainly rely on fatty acid oxidation to meet the energy demands for pumping blood and that the energy supply from glucose significantly increased after I/RI. Additionally, the diabetic myocardium mainly depends on the metabolism of fatty acids, especially after RI; the diabetic myocardium is overly dependent on fatty acids for energy supply, and glucose utilization is significantly lower than that in healthy myocardium and may be the main cause of myocardial vulnerability. This finding verified the scRNA‐seq analysis results and reinforced the theoretical speculation that diabetic myocardial metabolic reprogramming causes increased myocardial vulnerability. The results indicated that the main causes of the over‐reliance on fatty acids as metabolic substrates after I/RI in diabetic myocardial tissues are the substantial reduction in the number of C6‐CMs in diabetic ventricular muscle and the effect of metabolic reprogramming caused by the heterogeneity of the C6‐CMs, which is completely consistent with the scRNA‐seq analysis results.

The scRNA‐seq results showed that the expression level of Txnip/Pdk4 was significantly increased in diabetic myocardium, indicating that Txnip/Pdk4 plays an important role in the reprogramming of energy metabolism in diabetic myocardium. To reconfirm the scRNA‐seq and metabolomics results that increased myocardial vulnerability is due to metabolic reprogramming in diabetic myocardium, mice were treated with TXNIP/PDK4 inhibitors, that is, verapamil [45] and DCA [46], respectively. After the administration of TXNIP/PDK4 inhibitors to diabetic mice after I/RI, glucose metabolism increased, and some fatty acid metabolism was inhibited, with protection from myocardial injury and protection of cardiac function, suggesting that improvements in diabetic myocardial energy metabolism significantly attenuate myocardial vulnerability.

TXNIP is a cytokine widely expressed during various cellular stresses, including ischemia, oxidative stress, and apoptosis. TXNIP binds to thioredoxin to inhibit its antioxidant activity and promotes oxidative stress to aggravate mitochondrial damage [47]. TXNIP expression inhibits glucose transport by Glut1 and Glut4 on the cardiomyocyte membrane [48], and TXNIP inhibits glycolysis by inhibiting the expression of Hif‐1α, downregulating the expression of key glycolytic enzymes, and indirectly promoting fatty acid metabolism through the Randle cycle [49]. Therefore, TXNIP regulates energy metabolism through multiple pathways and is considered a switch that regulates energy metabolism in the process of myocardial I/RI [50]. In this study, the L‐type calcium channel blocker verapamil was used to inhibit TXNIP [51] and effectively reduce the synthesis of TXNIP in heart tissue, thereby regulating energy metabolism and reducing the over‐reliance on fatty acids. TXNIP affects myocardial energy metabolism in the long term through several pathways, and in this study, TXNIP was also found to play an important role in the formation and development of diabetic myocardium. The results of this study showed that after feeding verapamil to db/db mice for 3 weeks, the body weight of db/db mice was significantly lower than that of normally fed db/db mice, indicating that the inhibition of TXNIP can not only attenuate the formation of diabetic myocardium but also resist myocardial I/RI, thus exerting a myocardial protection effect.

The competition between fatty acids and glucose in myocardial tissue occurs at the pyruvate dehydrogenase complex (PDC) level, and pyruvate dehydrogenase kinase (Pdk) can phosphorylate PDC to inhibit its activity [52]. Currently, four PDK isoenzymes have been identified in humans and rodents, that is, PDK1, PDK2, PDK3, and PDK4, and each isoform has a tissue‐specific distribution, with PDK4 predominantly found in high‐energy‐demanding and mitochondria‐rich tissues, including skeletal muscle, myocardium, liver, and vascular tissues [53]. DCA is an inhibitor of PDK4 [54]. In this study, DCA was administered 15 min before ischemia. The results showed that DCA promoted glucose utilization after I/RI, indicating that DCA can rapidly adjust myocardial energy metabolism, thus improving myocardial vulnerability after I/RI.

After TXNIP/PDK4 inhibitor treatment, the myocardial vulnerability of the diabetic myocardium after I/RI was greatly reduced, exerting a protective effect on myocardial tissues, indicating that fully optimizing glucose metabolism in the diabetic myocardium has multiple benefits. Consistent with previous studies [55, 56], this study found that TXNIP played an important role in the formation of diabetic myocardium, that the inhibition of TXNIP expression could slowly and continuously improve the function of diabetic myocardium, that the inhibition of PDK4 could rapidly optimize energy metabolism in diabetic myocardium, and that the simultaneous use of the two can act synergistically to achieve a better effect.

However, this study has the following limitations. (1) Due to the small volume of the mouse heart, metabolomics of the ventricular muscle alone cannot be performed; although the mouse heart is mainly composed of the ventricle and the auricles were intentionally removed at the time of harvesting, there was still some atrial tissue, which may have affected the results. (2) The main purpose of the TXNIP/PDK4 intervention was to explore the possibility of clinical translation; therefore, gene knockout modeling was not performed.

## 4 Conclusion

The aim of this study was to address the practical issue of increased vulnerability after diabetic myocardial I/RI. scRNA‐seq revealed that the reduced number and functional heterogeneity of C6‐CMs subtypes were the material basis of increased vulnerability. FISH verified the substantial reduction in the number of C6‐CMs following diabetic myocardial I/RI and localized C6‐CMs to ventricular myocardial tissue. Metabolomics verified that over‐reliance on fatty acid metabolism increased myocardial vulnerability after diabetic myocardial I/RI. The optimization of glucose metabolism through TXNIP/PDK4 intervention can exert a myocardial protection effect and thus has potential clinical application value. This study proposed for the first time that different subtypes of cardiomyocytes have their own unique functional characteristics, and this heterogeneity plays an important role in different pathological states. It will open up new ideas for future research on various heart diseases.

## 5 Experimental Section

### 5.1 Animal Experiment

8–10‐week‐old male C57BL/6J mice and male db/db mice were selected respectively (animals were provided by Changzhou Cavinston Laboratory Animal Co. Ltd. in Jiangsu, China). Each group contains 3 C57BL/6J mice, and the samples are mixed for single‐cell nuclear sequencing. All experiments involving animals were performed in accordance with the *Guidelines for the Use and Care of Experimental Animals*. The animal protocols were approved by the Animal Care and Use Committee and Institutional Review Committee of The First Affiliated Hospital of Xinjiang Medical University (approval number: 230307‐79).

### 5.2 Experimental Grouping

Healthy C57BL/6J mice were divided into sham‐operated (NM) and C57BL/6J I/RI mice (NMI); diabetic db/db mice were divided into sham‐operated (DM) and diabetic db/db I/RI mice (DMI).

### 5.3 Experimental Modeling

Experimental mice were anesthetized with 1.5% sevoflurane and fixed on a rat table after complete disappearance of eyelid and foot reflexes. Laryngoscopy was performed using a 17^#^ trocar needle for tracheal intubation, and mice were mechanically ventilated using a small animal ventilator. Tidal volume was 30–50 mL/kg, respiratory rate was 60 breaths/min, inspiratory–expiratory ratio = 1:2, and FiO_2_ was 99%. Anal temperature was maintained at 37°C with an animal insulation blanket, and heart rate was continuously monitored using a biosignal acquisition and processing system. An incision was made in the 3rd or 4th intercostal space at the left edge of the sternum. The single layer of chest wall muscle was then separated directly to access the chest cavity, and the pericardium was incised to expose the heart. The I/RI group (30 min of ischemia and 120 min of reperfusion) was prepared by ligating the left anterior descending coronary artery (LAD) with a 6/0 filament approximately 2 mm below the root of the left auricle. Myocardial ischemia was confirmed by localized epicardial cyanosis below the level of ligation and ST‐segment elevation on the electrocardiogram; the sham‐operated group was threaded only without ligation. Mice were executed at 120 min of reperfusion, and myocardial tissue was removed and stored at −80°C in a refrigerator for subsequent sequencing. After study, all animals were anesthetized with an intraperitoneal injection of sodium pentobarbital (50 mg/kg body weight) and then euthanized by cervical dislocation.

### 5.4 Experimental Procedure

After completion of the maneuvers according to the ischemia–reperfusion modeling or sham procedure, whole hearts were taken after euthanasia for cervical dislocation. Blood was removed from the heart by immediate DPBS perfusion from the mouse aorta; cardiac tissue was collected and placed in liquid nitrogen precooled centrifuge tubes, and the heart tissue was frozen in liquid nitrogen for 1–2 h. Cardiac tissue was pulverized into 1–3‐mm pieces, and 10–20 pieces of cardiac tissue were randomly collected for nucleation. The samples were transferred to cryotubes, snap‐frozen in liquid nitrogen, and stored at −80°C until processing. Sequencing was performed by Shanghai Boho Biologicals Co. Ltd. (Shanghai, China) on a 10xGenomics system (Carlsbad, CA, USA) (MORE detailed experimental methods, equipment, reagents, and data can be found in Appendix S1).

### 5.5 Statistical Methods

All data are expressed as the mean ± standard deviation ($\bar{x}\pm s$). The difference between two groups was analyzed using Student's *t*‐test, the comparison of three groups was performed using one‐way analysis of variance (ANOVA), and continuous data were analyzed using repeated measures ANOVA. *p* < 0.05 was considered statistically significant.

## Author Contributions

**Zheng Zeng:** investigation, validation, visualization, animal experiments, writing – review and editing. **Wei Shi:** resources, project administration, supervision, writing – review and editing. **Yuxuan He:** methodology, software, data curation, formal analysis. **Yue Wang:** investigation, validation, metabolomics. **Cong Pu:** supervision, funding acquisition. **Jingtong Tan:** FISH experiments. **Hai Guo:** writing – review and editing. All authors critically revised the manuscript, approved the final version, and agree to be accountable for all aspects of the work in accordance with the latest ICMJE guidelines. **Haiping Ma:** conceptualization, methodology, investigation, data curation, formal analysis, writing – original draft, funding acquisition.

## Funding

This work was supported by Outstanding Talent Program of the First Affiliated Hospital of Xinjiang Medical University (ZYRC202402) and Xinjiang Strategic Talent Development Plan – Specialized Talent Project for Urgent Needs (XJRC‐2025‐RS‐PY‐TX‐035).

## Conflicts of Interest

The authors express their deep appreciation for Dr. Fen Liu from The Animal Laboratory Center of Xinjiang Medical University and the team of Dr. Xing Zhang from The Fourth Military Medical University. The authors declare no conflicts of interest.

## Supporting information


**Appendix S1:** jdb70277‐sup‐0001‐AppendixS1.docx.

**Appendix S2:** jdb70277‐sup‐0002‐AppendixS2.docx.

**Appendix S3:** jdb70277‐sup‐0003‐AppendixS3.docx.

**Appendix S4:** jdb70277‐sup‐0004‐AppendixS4.docx.

**Appendix S5:** jdb70277‐sup‐0005‐AppendixS5.doc.

**Appendix S6:** jdb70277‐sup‐0006‐AppendixS6.docx.

**Appendix S7:** jdb70277‐sup‐0007‐AppendixS7.docx.

## Data Availability

The data that support the findings of this study are available on request from the corresponding author. The data are not publicly available due to privacy or ethical restrictions.
