# Supplementary material for: Txnip/PDK4‐Mediated Hyperdependence on Fatty‐Acid Oxidation Amplifies Susceptibility of Diabetic Myocardium to Ischemia–Reperfusion Damage
Source: J Diabetes. 2026 Oct 20;18(10):e70277. doi: 10.1111/1753-0407.70277 (PMC13681344; doi:10.1111/1753-0407.70277)
Supplement: Supplementary file 1 [file JDB-18-e70277-s001.docx]

*Appendix 7.*

**Uncropped Western Blot Exposure Image**

We have compiled all uncropped exposures of Western blot immunoblots from the manuscript.

 Figure 1.TXNIP uncropped Western Blot Exposure Image

 Figure 2.PDK4 uncropped Western Blot Exposure Image

 Figure 3.HK2 uncropped Western Blot Exposure Image

Figure 4.LDH uncropped Western Blot Exposure Image

Figure 5.PFK uncropped Western Blot Exposure Image

Figure 6.PKM uncropped Western Blot Exposure Image

Figure 7.ACSL1 uncropped Western Blot Exposure Image

Figure 8.ACOX uncropped Western Blot Exposure Image

Figure 9.CPT-1 uncropped Western Blot Exposure Image

Figure 10.PPARα uncropped Western Blot Exposure Image

Figure 11.Actin uncropped Western Blot Exposure Image
