# Supplementary material for: Txnip/PDK4‐Mediated Hyperdependence on Fatty‐Acid Oxidation Amplifies Susceptibility of Diabetic Myocardium to Ischemia–Reperfusion Damage
Source: J Diabetes. 2026 Oct 20;18(10):e70277. doi: 10.1111/1753-0407.70277 (PMC13681344; doi:10.1111/1753-0407.70277)
Supplement: Supplementary file 2 [file JDB-18-e70277-s002.docx]

*Appendix 6.*

**Myocardial metabolomics analysis**

Metabolomics can identify and quantify multiple metabolites through mass spectrometry combined with gas chromatography or liquid chromatography (GC/LC-MS). A study was conducted on 4 groups of samples (NM, NMI, DM, and DMI), with each group repeating 5 times and extracting 16764 Peaks from a total of 20 experimental samples. In order to reduce the impact of detection system errors on the results and better highlight their biological significance, a series of preparations and organization were carried out on the original data. The main steps include: deviation value filtering, missing value filtering, missing value filling, and data standardization processing. Normalize the detection data according to the internal standard. After the above preprocessing, 11085 Peaks were ultimately retained in this project (see Table 1), including 4466 differentially expressed metabolites, of which 2862 were downregulated; Lowered 1604.

**Table 1 Summary table of metabolites detected by non-targeted metabolomics**

| Group | Cpd_all | Cpd_diff | Cpd_diff_up | Cpd_diff_down |
| --- | --- | --- | --- | --- |
| NM vs DM | 11085 | 1447 | 812 | 635 |
| NMI vs DMI | 11085 | 1133 | 878 | 255 |
| NM vs NMI | 11085 | 745 | 318 | 427 |
| DM vs DMI | 11085 | 1141 | 854 | 287 |

The study was based on the LC-MS/MS quadrupole electrostatic field orbital trap Orbitrap mass spectrometer (Thermo Fisher Scientific, USA) for detection and analysis. A total of 815 metabolites were detected, classified and accounted for: alkaloids and their derivatives (0.3%), benzene ring type (5.79%), homogeneous non-metallic compounds (0.61%), hydrocarbons (0.3%), lignans, neolignans and related compounds (0.3%), lipids and lipid molecules (32.93%), nucleosides, nucleotides and analogues (7.62%), organic acids and their derivatives (21.04%), and organic nitrogen compounds (5.18%). 14 types of compounds, including organic oxygen compounds (8.84%), organic heterocyclic compounds (11.89%), organic sulfur compounds (0.61%), others (0.61%), and phenylpropanoids and polyketones (3.96%); Among them, the proportion of lipid and lipid molecular metabolites is the highest (see Figure 1), clustered into 6 families (see Figure 2). The proportion of various metabolites mentioned above clearly indicates that lipids are the main substrates for myocardial energy acquisition.

Figure caption: Different colored blocks in the figure represent different classification categories, and the percentage represents the percentage of metabolites belonging to that type among all identified metabolites.

**Figure 1 Donut Plot of metabolite classification and proportion**

**Figure 2 Clustering heat maps of four groups of metabolites**

Through screening of substrates for fatty acid and glucose metabolism, a total of 48 metabolites were detected (see Figure 3), and 18 differential metabolites were screened (Table 2 and Figure 4); Among them, 13 were upregulated and 5 were downregulated. Through the metabolite clustering heat map (Figure 5), it can be clearly found that the number and expression of fatty acid metabolites in DM group and DMI group are significantly higher than those in NM group, indicating that diabetes myocardium relies more on fat metabolism to obtain energy, and the results again verify the previous theoretical speculation.

**Figure 3 Clustering heat maps of differences in fatty acid and glucose metabolism in all samples**

Figure caption: A represents the Venn diagram of four differential metabolites; B is a volcano plot showing the differential metabolites between the NMI group and the DMI group; Each point in the volcano map represents a peak, which includes all the substances measured in this experiment.

**Figure 4 Venn diagram of differential metabolites and NMI/DMI volcano diagram**

**Table 2 Summary of differential metabolites**

| Group | Cpd_all | Cpd_diff | Cpd_diff_up | Cpd_diff_down |
| --- | --- | --- | --- | --- |
| DM vs DMI | 48 | 7 | 6 | 1 |
| NM vs DM | 48 | 3 | 1 | 2 |
| NMI vs DMI | 48 | 6 | 5 | 1 |
| NM vs NMI | 48 | 2 | 1 | 1 |

Through the comparison of glucose and fatty acid metabolism foreign bodies in healthy mice (NM group) and diabetes mice (DM group), it was found that there were four kinds of foreign bodies, including propionyl carnitine, Sedum heptanose, (R) − 3 − hydroxy − myristic acid and adrenal acid. Propionyl carnitine, also known as C3 hydroxybutyrate, is a coenzyme in fat metabolism that can be synthesized through the action of acetyl CoA carboxylase. Propionyl C3 hydroxybutyric acid in the human body is usually synthesized from fatty acids and is mainly metabolized in the liver. It is an important enzyme in energy metabolism and can synthesize fat through the action of acetyl CoA carboxylase. The high expression of this metabolite in diabetes myocardium (DM group), while Sedum Sedum heptosaccharide, (R) − 3 − hydroxy − myristic acid and adrenal acid in healthy myocardium are much higher than those in diabetes group. Both groups have high expression of intermediate products of fatty acid metabolism, which may be related to cardiomyopathy's ability to metabolize fatty acid; However, the high expression of Sedum Sedum Gentose in NM group indicates that glucose utilization in healthy myocardium is higher than that in diabetes group; Compared with NMI/DMI, in addition to the high expression of organic acid 3 − hydroxyvaleric acid in NMI, five lipid metabolites (octanedioic acid, dodecanedioic acid, hexanoic acid, 12 − hydroxystearic acid and octadecanedioic acid) are highly expressed in the DMI group, indicating that the myocardium is mainly powered by fatty acid metabolism, and a small part of glucose is powered by healthy myocardium, but the glucose metabolism of diabetes myocardium is significantly reduced. After the occurrence of I/RI in diabetes myocardium, it is more obvious to rely on fatty acid metabolism to obtain energy.

**Figure5 Cluster heat maps of fatty acid and glucose metabolites between groups**

**3 Glucose Metabolic Flow Analysis**

In order to study the contribution ratio of glucose to TAC in the four groups under different conditions, metabolic flow analysis was carried out on U-13C labeled glucose metabolism difference foreign bodies. The results showed that the differential metabolites in the glycolysis process of glucose, such as G6P, F6P, FBP, G3P, PEP, LAC and PYR, were significantly higher in the NM group than in the DM group. After I/RI, the glycolysis of healthy myocardium was enhanced, and the glycolysis process of diabetes myocardium had little change, while the NMI was significantly higher than that of DMI (P<0.05); The differential metabolites during the TCA cycle, such as citrate (CIT), isocitrate (ICIT), a-ketoglutaric acid (a-KG), succinate (SUC), fumarate (FUM), and malate (MALE), were significantly reduced in the NM group compared to the NMI group and the DM group compared to the DMI group (P<0.05); It is evident from the metabolite clustering heatmap that the metabolites of glucose in the NMI group are significantly higher than those in the DMI group. Although there was a sample in the NMI group (NMI2) with low expression of all glucose metabolites, the analysis was due to the failure of U-13C glucose injection during the modeling process, but it did not have a significant impact on the overall statistical results. The results showed that the glycolysis of healthy myocardium was significantly enhanced after I/RI, while the glycolysis of diabetes myocardium was not significantly changed after I/RI. Compared with diabetes myocardium, the glycolysis of healthy myocardium was significantly increased after I/RI; Moreover, the expression level of glucose metabolites in healthy myocardium during TCA circulation is much higher than that in diabetes myocardium, and diabetes myocardium will rely more on fatty acid for energy supply after Randle circulation (see Fig. 6A and 6B).

Figure 6A.Diagram of U-^13^C as a tracer ;Figure6 B.Cluster heatmap of differential metabolites.

**Figure** **6 U-^13^C labeled glucose as tracer and clustering heat map**

In conclusion, U-13C labeled glucose metabolic flow analysis showed that the ability of healthy mice to use glucose as an energy substrate was significantly higher than that of diabetes mice. The glycolysis metabolism of healthy myocardium was significantly increased after I/RI, while the glycolysis of diabetes myocardium did not change significantly after I/RI. The glycolysis process of healthy myocardium after I/RI was significantly higher than that of diabetes myocardium; Similarly, the expression level of glucose metabolites in healthy myocardium during TCA circulation is much higher than that in diabetes myocardium, and diabetes myocardium will rely more on fatty acid for energy supply after Randle circulation. The result once again more accurately validated the previous scientific hypothesis.

Table 3. Changes in body weight and blood glucose in db/db mice after 3 weeks of verapamil inhibition of TXNIP

|  | Experimental group (n=5) | | Control group (n=5) | | *P* value |
| --- | --- | --- | --- | --- | --- |
|  | Before intervention | After intervention | Before intervention | After intervention |  |
| Blood glucose (mmol/L) | 6.30±3.53 | 16.82±3.320 | 6.36±3.23 | 24.68±6.25 | <0.001 |
| Weight (g) | 24.80±2.69 | 31.60±2.15 | 24.54±1.94 | 38.78±1.75 | <0.001 |

Changes in blood glucose and body weight in mice before and after administration of TXNIP inhibitors.

Table 4.Comparison of cardiac ultrasound data in each group

|  | HR (beats/min) | SV (ml/beats) | CO (L/min) | EF (%) | FS (%) |
| --- | --- | --- | --- | --- | --- |
| NM | 333.67±63.96 | 38.01±14.82 | 13.39±5.74 | 66.28±2.90 | 35.77±1.64 |
| NMI | 377.95±17.71 | 28.09±10.79 | 10.71±4.46 | 42.14±3.71 | 20.36±2.31 |
| DM | 377.07±56.01 | 30.72±12.22 | 11.68±4.98 | 54.24±2.11 | 27.40±1.35 |
| DMI | 394.83±44.15 | 29.58±8.13 | 11.89±4.14 | 29.81±5.62 | 13.96±2.76 |
| D+DMI | 356.91±36.77 | 30.90±6.97 | 11.07±3.11 | 47.15±1.61 | 23.25±0.96 |
| V+DMI | 429.94±32.25 | 28.02±3.09 | 12.03±1.47 | 40.35±3.37 | 19.39±1.87 |
| D/V+DMI | 428.04±35.82 | 37.73±6.31 | 16.01±2.00 | 49.27±2.92 | 24.66±1.85 |
| *P* value | 0.004 | 0.344 | 0.310 | <0.001 | <0.001 |

Heart rate, stroke output, cardiac output, ejection fraction, and long axis shortening rate of each group of mice.
