# Supplementary material for: Txnip/PDK4‐Mediated Hyperdependence on Fatty‐Acid Oxidation Amplifies Susceptibility of Diabetic Myocardium to Ischemia–Reperfusion Damage
Source: J Diabetes. 2026 Oct 20;18(10):e70277. doi: 10.1111/1753-0407.70277 (PMC13681344; doi:10.1111/1753-0407.70277)
Supplement: Supplementary file 3 [file JDB-18-e70277-s004.docx]

*Appendix 3.*

**Screening of target genes**

By analyzing the trend of the number of cardiomyocyte subsets, it was found that the C6 cardiomyocyte subsets, which accounted for the largest proportion (42.1%) in normal myocardium, were significantly reduced in the course of diabetes and IR/I.Then, the DEGs of C6 cardiomyocyte subgroup was analyzed, and it was found that the main function of C6 cardiomyocyte subgroup was concentrated in energy metabolism and contractile function, which was relatively independent from other types of cardiomyocyte subgroups in function, and it was considered that C6 subgroup was mainly responsible for myocardial contractile function.

Then, the energy metabolism characteristics of the C6 subgroup were analyzed by bioinformatics and data mining, and it was found that there was significant heterogeneity in energy metabolism of the C6 subgroup of cardiomyocytes. In particular, the excessive dependence of diabetic cardiomyocytes on fatty acid oxidation may be a special attribute leading to increased myocardial I/RI vulnerability.

Comprehensive speculation: Because the number of C6 myocyte subsets, which are mainly responsible for systolic function, is significantly reduced in diabetic myocardium;C6 cardiomyocyte subsets have significant heterogeneity in energy metabolism. In particular, the excessive dependence of diabetic cardiomyocytes on fatty acid oxidation may be its special attribute. Combined with the above two points, it may be the material basis for the increased vulnerability after diabetes I/RI.Therefore, C6 subgroup was identified as the focus subgroup of this study, and target genes that play a leading role in regulating energy metabolism under different states were screened.

The reasons for the increased myocardial vulnerability of diabetic IR/I are both "diabetes" and "I/RI" factors. Therefore, the screening principle of target genes should take into account both the causes of IR/I and the pathophysiological changes of diabetes (as shown in the diagram below).

①IR/I is an acute and transient process, which involves more damaging factors, so it can be used as a clue to screen differential genes.The screening criteria are:LOGFC>0.5;P-value<0.05.The specific screening process is as follows:according to DEGs analysis, 195 genes of normal IR/I were up-regulated, while 256 genes of diabetic IR/I were up-regulated.The "pheatmap" R package analysis showed that there were 229 up-regulated genes specific to diabetes.According to the difference of the mean expression of IR/I after diabetes as the standard, the unique up-regulated genes of diabetes were rearranged and the top 20 genes were drawn according to four experimental states.PDK4 was selected as a gene involved in the regulation of myocardial energy metabolism (see Figure 1-2).

*Figure 1. DEGs Venn diagram*	*Figure 2. Heatmap of DEGs*

②In this study, diabetes mellitus is another important factor contributing to increased myocardial IR/I vulnerability.Since previous screening focused more on acute changes in IR/I, the diabetes factor focused more on a long-term, chronic pathophysiological change in diabetic myocardial formation.Screening criteria:|LOGFC|>0.5;P-value<0.05.The process is as follows:DEGs analysis was performed on diabetic and normal IR/I and diabetic and normal IR/I respectively.Compared with normal mice, 247 up-regulated genes and 107 down-regulated genes were found in diabetic mice.Compared with normal IR/I mice, 1192 genes were up-regulated and 114 genes were down-regulated in diabetic IR/I mice.The "pheatmap" R package analysis showed that 48 genes had pathological changes due to diabetic myocardial disease, while no changes were found in IR/I. According to the absolute value of the difference between diabetes and normal transformation, the 48 genes were rearranged and the top 20 genes were drawn according to four experimental states.TXNIP was screened as an important gene involved in the regulation of myocardial energy metabolism (see Figure 3-6).

*Figure 3.DM vs NM DEGs Volcano Plot	Figure 4. DMI vs NMI DEGs Volcano Plot*

*Figure 5. DEGs Venn diagram	Figure 6. Heatmap of DEGs*

*Figure 3*.Volcano map showing differential genes between DM and NM groups;

*Figure 4*.The volcano plot shows the differential genes between the DMI and NMI groups;

*Figure 5*. Wayne plot shows the upregulated genes between the DM and NM groups, DMI and NMI groups, NMI and NM groups, and DMI and DM groups;

*Figure 6*. heatmap shows the relative expression levels of genes between the groups.

In order to conduct in-depth analysis on the change characteristics of Txnip and Pdk4 among the four groups, the broken line chart (Figure 7) shows that the change characteristics of Txnip are: NM is higher than NMI and DM than DMI, and this change is not significant, but DM is significantly higher than NM, indicating that diabetes itself has a greater impact on Txnip, so Txnip is a representative gene that mainly reflects the impact of diabetes pathological changes; Pdk4 is up-regulated after myocardial I/RI (NMI/DMI) in health and diabetes, but the increase is most significant after myocardial I/RI (DMI) in diabetes, so it is a representative gene that mainly reflects the impact of I/RI (Figure 7). This result is consistent with the above screening results.

*：*P*＜0.05；**：*P*＜0.01；***：*P*＜0.001。

**Figure 7 Transcriptional level analysis of Txnip and Pdk4**

In summary, TXNIP and PDK4 are selected as the target genes of this study, which will be further analyzed and studied in the next step.
