# Supplementary material for: Txnip/PDK4‐Mediated Hyperdependence on Fatty‐Acid Oxidation Amplifies Susceptibility of Diabetic Myocardium to Ischemia–Reperfusion Damage
Source: J Diabetes. 2026 Oct 20;18(10):e70277. doi: 10.1111/1753-0407.70277 (PMC13681344; doi:10.1111/1753-0407.70277)
Supplement: Supplementary file 4 [file JDB-18-e70277-s005.docx]

*Appendix 2.*

**Functional analysis of the C0 subpopulation**

This study identified a total of 26 clusters, annotated as 11 different cell lineages. These included 5 cardiomyocyte subpopulations (C0, C3, C4, C6, C11). Analysis of changes in the proportion of cardiomyocyte numbers revealed that: 29.6% of cardiomyocyte subpopulations in the NM group, 24.4% in the NMI group, 44.2% in the DM group, and 54.4% in the DMI group, indicating that the proportion of cardiomyocytes in the DM group was significantly higher than that of the NM group, and that cardiomyocytes were significantly decreased in the NM group compared to the NMI group, while they were significantly increased in the DM group compared to the DMI group (Fig.1, Fig.3). Among the cardiomyocyte subpopulations, the change in the percentage of C0 subpopulation numbers was the most specific (Fig.2), which resulted in a significant increase in the number of cardiomyocytes in diabetes as well as in diabetic IR/I cardiomyocytes instead of a decrease due to an abnormal increase in the number of C0 cardiomyocyte subpopulation in diabetes.

Fig. 1 TSNE plot of the proportion of cell number in each group

Fig.2 TSNE plot of the proportion of C0 in the number of cells in each group

**The percentage of C0 subpopulation numbers was analyzed**

The number of C0 subpopulations accounted for 2.4% in the NM group, 6.5% in the NMI group, 16.7% in the DM group, and 41.9% in the DMI group, suggesting that the occurrence of IR/I can all lead to a significant increase in the number of C0 subpopulations, with the most significant increase in diabetes, where it has become the most predominant cardiomyocyte constituent of the cardiomyocytes, with a proportion of almost one-half, in the diabetic IR/I group. This significant change in number may account for the fact that the number of cardiomyocytes did not decrease, but rather increased, after diabetic myocardial IR/I (Fig.2,Fig.5). Certainly, substantial changes in the C0 cardiomyocyte subpopulation of cells will certainly lead to functional alterations and need to be explored in depth.

Fig. 3 Proportion of cell number in each group Fig.4 Analysis of cardiomyocyte subpopulation occupancies

Fig.5 Analysis of the percentage of C0 cardiomyocyte number

**Heterogeneity analysis of C0 cardiomyocyte subpopulations**

The UMAP of C0 cardiomyocyte subpopulation and other cardiomyocyte subpopulations was demonstrated by Louper software (Fig. 6); the globally of loupe software was used to calculate the differential genes of the five groups of C0, C3, C4, C6, and C11, and the csv files of all genes added by the five subtypes of LOG2FC were exported, and the genes labeled with an asterisk in the loupe software were selected as the up-regulation of the differential genes to draw the heat map of the C0 subgroup genes (Fig. 7), and the obtained differential genes were used to draw the Wein diagrams using two packages of R's venn and VennDiagram (Fig. 8), and the comprehensive analysis found that the C0 cardiomyocyte subgroups were functionally closer to the C3, C4, and C11 subgroups, and were more different from the C6 subgroups;The number of DEGs was low (61) and most of them intersected with other subpopulations (52), suggesting that C0 is functionally an insufficiently characterized cardiomyocyte subtype. However, its characteristics are relatively close to those of C3, C4, and C11, which are more different from the C6 subpopulation in terms of characteristics. Since C3, C4, and C11 have been previously analyzed and identified as, ventricular junction zone, atrial myocardium, and sinoatrial node cells, it suggests that C0 is functionally a cardiomyocyte not characterized by contraction.

Fig.6 UMAP of the C6 subpopulation	Fig.7 Heat map of C6 subpopulation genes

Fig.8 Wayne diagram of cardiomyocytes

To continue the in-depth analysis of the ambiguous functional characteristics of the C0 subpopulation, a small lift diagram of endothelial cell marker genes (Flt1, Adgrf5, Cdh5 and Pecam1) in the C0 cardiomyocyte subpopulation was drawn by Louper software, which revealed that the expression levels of the endothelial cell genes (Tnnt2, Ryr2, and Myh6) in the C0 subpopulation were significantly higher than that in the other subpopulations of cardiomyocytes (Fig. 9).The most significant cardiomyocyte marker genes (Tnnt2, Ryr2 and Myh6) were expressed in all five cardiomyocyte subpopulations (Fig.10), suggesting that the C0 subpopulation had both endothelial cell genes and cardiomyocyte genes, and it was a kind of transitional cell between cardiomyocytes and endothelial cells, so it was thought to be a possible endothelial cardiomyopathy-like altered cell.

Fig. 9 Violin map of marker genes in endothelial cells of C0 subpopulation

Fig.10 Violin map of marker genes in C0 subpopulation cardiomyocytes

The KEGG and GO analyses of the DEGs of C0 cardiomyocytes, as the cardiomyocyte subpopulation with the most significant change in the number of percentage, found that the main focus was on the influence of signaling pathways, such as protein paracrine, adhesion, and vascular proliferation, etc. The analyses of the DEGs of the C0 subpopulation between the two groups of NM and DM found that C0 was weakened in the diabetic group (Fig.11). The main functions of C0 were autophagy/metabolism/contraction/anti-oxidative stress (Figs.11,12). Myocardial microangiopathy is the main pathological change in diabetic cardiomyopathy, and diabetes mellitus is closely related to the value-added and pathological generation of microvessels.This trend of cell number change in C0 subpopulation is in line with the characteristics of pathological changes in diabetic cardiomyopathy, and this pathological change supports that the endothelial cells have the potential to be transdifferentiated into cardiomyocytes in the presence of certain specific microenvironmental stimuli such as hypoxia. Therefore, it is believed that the C0 subpopulation is a special atypical cardiomyocyte type, which needs to be further analyzed.

Fig.11 KEGG analysis of TOP20 gene of C0	Fig.12 GO analysis of TOP20 genes of C0

**Analysis of C0 subgroups among the four groups**

Changes in the proportions of different cell types in the four samples showed that endothelial cells were the most numerous cell type among cardiac mesenchymal cells. The number of endothelial cells was significantly lower in the NMI than in the NM group, and further decreased in the DMI group compared to the DM group, while C0 cardiomyocytes increased significantly instead (Fig.13). The decreasing trend shown in the percentage of endothelial cell number and the increasing trend in the percentage of C0 cardiomyocytes reaffirmed the inference of endothelial cell to cardiomyocyte transformation.

Fig. 13. Changes in the proportion of different cell types under different conditions

In recent years, it has been found that endothelial cells exhibit a unique multidirectional differentiation capacity when stimulated by physiological or pathological factors such as hyperglycemia, hypoxia, inflammation, and abnormal lipid metabolism. It has been shown that each cardiomyocyte is closely aligned with the endothelial cells, and that this close anatomical structure within the coronary microvascular network not only allows for adequate blood supply but also facilitates cell-cell interactions. It has been found that mature cardiomyocytes appear to be derived not only from a single lineage but some from transdifferentiation of endothelial cells, and cardiomyocytes derived from transdifferentiation of endothelial cells have been found in ischemia-modeled mice and elaborated on the involvement of endothelial cells in postischemic cardiomyogenesis [1,2]. Another study also reported that adult blood-derived endothelial progenitor cells transdifferentiated into functionally active cardiomyocytes and showed characteristic phenotypes of cardiomyocytes as well as kinetic properties [3]. Transdifferentiation is usually verified by transient co-expression of markers for two different cell types in the same cell, a phenomenon that indicates the transition of cells from one state of differentiation to another.

**Analyze DEGs of subgroup C0**

GSEA analysis of C0 subpopulation DEGs revealed that genes enriched more in adhesion, chemokine signaling pathway, PI3K-Akt signaling pathway, tight junction, and endocytosis (Fig. 14). The C0 subpopulation was found to have higher levels of protein synthesis and secretion, and the expression of HS-GAG contributed to fibroblasts value-added to play a role in promoting vascular fibrosis. Endothelial cells have strong paracrine regulation and can secrete a variety of endothelial-derived bioactive substances and vascular regulatory factors, which play an important role in the dynamic maintenance of vascular tone and angiogenesis [4-5]. The original cardiomyocytes do not play a prominent role in this regard, and their functions are mainly reflected in contraction, autoregulation and conduction, so it is inferred that the C0 subpopulation has endothelial cell characteristics.

Fig.14 GSEA analysis and gene heatmap of the C0 subpopulation

Therefore, the comprehensive analysis suggests that the C0 subpopulation, as an endothelial cell type, is a special type of cell formed under a variety of pathological changes, which participates in the process of diabetic myocardiogenesis and plays an important role in the increase of myocardial vulnerability due to diabetic myocardial IR/I. However, the mechanism of C0 subpopulation formation in diabetic myocardium as well as during IR/I still needs to be further explored and investigated.

**Reference:**

[1] Condorelli G, Borello U, De Angelis L, et al. Cardiomyocytes induce endothelial cells to trans-differentiate into cardiac muscle: implications for myocardium regeneration. Proc Natl Acad Sci U S A. 2001 Sep 11;98(19):10733-8.

[2] Ramanujam D, Schön AP, Beck C, et al. MicroRNA-21-Dependent Macrophage-to-Fibroblast Signaling Determines the Cardiac Response to Pressure Overload. Circulation. 2021 Apr 13;143(15):1513-1525.

[3] 柯伟良,李上海,王丹丹.成人血源性内皮祖细胞转分化为有功能活性的心肌细胞.中外医学研究.2015(01):153-155

[4] Hind LE, Ingram PN, Beebe DJ, et al. Interaction with an endothelial lumen increases neutrophil lifetime and motility in response to P aeruginosa. Blood. 2018 Oct 25;132(17):1818-1828.

[5] Xu S, Ilyas I, Little PJ, et al. Endothelial Dysfunction in Atherosclerotic Cardiovascular Diseases and Beyond: From Mechanism to Pharmaco- therapies. Pharmacol Rev. 2021 Jul;73(3):924-967.
