# Supplementary material for: Txnip/PDK4‐Mediated Hyperdependence on Fatty‐Acid Oxidation Amplifies Susceptibility of Diabetic Myocardium to Ischemia–Reperfusion Damage
Source: J Diabetes. 2026 Oct 20;18(10):e70277. doi: 10.1111/1753-0407.70277 (PMC13681344; doi:10.1111/1753-0407.70277)
Supplement: Supplementary file 5 [file JDB-18-e70277-s006.docx]

*Appendix 1.*

**Experimental methodology and quality control of single-cell nuclear sequencing**

**1.1 Laboratory animals**

8-10-week-old male C57 mice and male db/db mice were selected respectively (animals were provided by Changzhou Cavinston Laboratory Animal Co. Ltd. in Jiangsu, China).Each group contains 3 C57 mice, and the samples are mixed for single-cell nuclear sequencing.

1.2 **Experimental grouping:**

Healthy C57 mice were divided into sham-operated (NM) and C57 I/RI mice (NMI); diabetic db/db mice were divided into sham-operated (DM) and diabetic db/db I/RI mice (DMI).

**1.3 Experimental modeling:**

1.3.1 Instruments and reagents

Surgical sutures, microsurgical instruments, XR300 small animal anesthesia machine, XKRM6240 small animal electrocardiograph (Shanghai XinSoft Information Technology Co., Ltd. ), small animal surgical lamp, sevoflurane (Shanghai AbbVie).

1.3.2 Modeling

Experimental mice were anesthetized with 1.5% sevoflurane and fixed on a rat table after complete disappearance of eyelid and foot reflexes. Laryngoscopy was performed using a 17^#^ trocar needle for tracheal intubation, and mice were mechanically ventilated using a small animal ventilator. Tidal volume was 30-50 ml/kg, respiratory rate was 60 breaths/min, inspiratory-expiratory ratio = 1:2, and FiO_2_ was 99%. Anal temperature was maintained at 37°C with an animal insulation blanket, and heart rate was continuously monitored using a biosignal acquisition and processing system. An incision was made in the 3rd or 4th intercostal space at the left edge of the sternum. The single layer of chest wall muscle was then separated directly to access the chest cavity, and the pericardium was incised to expose the heart.The I/RI group (30 minutes of ischemia and 120 minutes of reperfusion) was prepared by ligating the left anterior descending coronary artery (LAD) with a 6/0 filament approximately 2 mm below the root of the left auricle. Myocardial ischemia was confirmed by localized epicardial cyanosis below the level of ligation and ST-segment elevation on the electrocardiogram; the sham-operated group was threaded only without ligation. Mice were executed at 120 min of reperfusion, and myocardial tissue was removed and stored at -80°C in a refrigerator for subsequent sequencing.After study, all animals were anaesthetized with an intraperitoneal injection of sodium pentobarbital (50 mg/kg body weight) and then euthanized by cervical dislocation.

**2.1 Experimental procedure**

After completion of the maneuvers according to the ischemia-reperfusion modeling or sham procedure, whole hearts were taken after euthanasia for cervical dislocation. Blood was removed from the heart by immediate DPBS perfusion from the mouse aorta; cardiac tissue was collected and placed in liquid nitrogen pre-cooled centrifuge tubes, and the heart tissue was frozen in liquid nitrogen for 1-2 h. Cardiac tissue was pulverized into 1-3-mm pieces, and 10-20 pieces of cardiac tissue were randomly collected for nucleation. The samples were transferred to cryotubes, snap-frozen in liquid nitrogen, and stored at -80°C until processing Sequencing was performed by Shanghai Boho Biologicals Co. Ltd (Shanghai, China) on a 10xGenomics system (Carlsbad, CA, USA).

2.1.1 Experimental materials for single cell suspension preparation

Table 1: Instrumentation for single-cell nuclear preparation

| **Brand name** | **Model number** | **Factory owners** |
| --- | --- | --- |
| 4°C centrifuge | Allegra X-15R | Beckman Coulter |
| cell counter | Countess™ II Automated Cell Counter | Thermo Fisher |
| fluorescence inverted microscope | CKX41 | Olympus |
| homogenizer | Vortex-6 | Kirin Bell (name) |
| thermostatic water bath | Shanghai Yiheng Technology Co. | Shanghai Yiheng Technology Co. |

Table 2: Consumables for single-cell nuclear preparation reagents

| **Brand name** | **Item number** | **Factory owners** |
| --- | --- | --- |
| Cell Nucleus Isolation Kit | 52009-10 | Shanghai Boho |
| 0.22μm /0.45μm Fliter 0.22μm /0.45μm Filter Membrane | -- | Millipore |
| 40 μm Cell Strainers (for cell filtration) | -- | -- |
| RiboLock RNase Inhibitor | EO038 | Thermo Scientific |
| DTT | - | - |
| BSA | - | - |

2.1.2 Quality control of single cell suspension preparation

Table 3 . List of experimental data

| **Sample name** | **Measured data G** | **Number of cells measured** | **Median number of genes** | **Average number of reads in the cell** | **Total reads** |
| --- | --- | --- | --- | --- | --- |
| NM | 104.65 | 13,435 | 1,332 | 25,964 | 348,826,445 |
| DM | 100.17 | 11,234 | 1,462 | 29,722 | 333,902,629 |
| NMI | 101.75 | 9,758 | 1,432 | 34,759 | 339,182,088 |
| DMI | 149.24 | 13,893 | 1,286 | 35,805 | 497,451,830 |

Figure 1. quality control analysis of nuclear suspension in NM group mice

Figure 2. Quality control analysis of nuclear suspension in DM group of mice

Figure 3. Quality control analysis of nuclear suspension in NMI group of mice

Figure 4. Quality control analysis of nuclear suspension in DMI group of mice

Table 4.Nuclear count

| **Sample Name** | **Concentration (pcs/μL)** | **Total number of nuclei** |
| --- | --- | --- |
| DM | 1290 | 645000 |
| NM | 958 | 479000 |
| NMI | 1050 | 842000 |
| DMI | 1090 | 109000 |

**2. 2 scRNA-seq experiments**


2.2.1 Experimental Materials

Table 5. scRNA-seq instrumentation

| **Brand name** | **Model number** | **Factory owners** |
| --- | --- | --- |
| Single Cell Sorting System | 10X Genomics Chromium | 10X Genomics |
| magnetic frame | 10X Magnetic Separator | 10X Genomics |
| PCR instrument | Veriti 96-Well Thermal Cycler | Thermo Fisher |
| homogenizer | Vortex-6 | Kirin Bell (name) |
| metal bath | Heat block | Major |
| Nucleic Acid Quantification Instrument | Qubit 4.0 Flourometer | Thermo Fisher |
| capillary electrophoresis | 2100 Bioanalyzer | Agilent |

Table 6. scRNA-seq reagent consumables

| **Brand name** | **Item number** | **Factory owners** |
| --- | --- | --- |
| Chromium Next GEM Chip G Single Cell Kit | 1000120 | 10X Genomics |
| Chromium Next GEM Single Cell 3' GEM, Library & Gel Bead Kit v3.1 | 1000121 | 10X Genomics |
| Chromium i7 Multiplex Kit | 120262 | 10X Genomics |
| SPRIselect Reagent Kit | B23318 | Beckman Coulter |
| Qubit Assay Tube | Q32856 | Thermo Fisher |
| ExKubit dsDNA HS | NGS00-3012 | ExCell Bio |
| High Sensitivity DNA Kit | 5067-4626 | Agilent |

2.2.2 Experimental Flow


2.2.2.1 10X Genomics onboarding and reverse transcription

(1) The prepared cell suspension, 10X barcode gel magnetic beads and oil were added to different chambers of Chromium Chip G to form GEM (Gel Beads-in-emulsion) via 10X Genomics Chromium system, respectively;

(2) GEM was transferred to a PCR instrument for reverse transcription, and gel magnetic beads containing 30 nt oligo-dT reverse transcription primers allowed poly-A RNA from cells to be reverse transcribed into a single strand of cDNA with Barcode and UMI information;

(3) Magnetic bead purification of one-stranded cDNA;

(4) The purified cDNA was subjected to PCR amplification;

(5) cDNA was assayed for concentration using a Qubit and fragment size using an Agilent 2100.

Table 7. cDNA quality control results

| **No.** | **Sample name** | **Concentration (ng/μL)** | **Main peak length (bp)** |
| --- | --- | --- | --- |
| 1 | D-M | 15.4 | 932 |
| 2 | N-M | 14.7 | 939 |
| 3 | NMI | 15.2 | 1457 |
| 4 | DMI | 12.5 | 1364 |

**The Agilent 2100 Bioanalyzer detection peaks are shown below:**

Figure 5. cDNA quality control analysis of NM group mice

Figure 6. cDNA quality control analysis of DM group mice

Figure 7. cDNA quality control analysis of mice in the NMI group

Figure 8. cDNA quality control analysis of mice in the DMI group

2.2.3 Sequencing library construction

(1) After the cDNA amplification is completed, the enzyme is sectioned and magnetic beads are screened for the most suitable fragments, and the cDNA library containing P5 and P7 junctions is constructed by PCR through end repair, addition of A, and junction ligation with Read2 sequencing primers.

(2) The library was purified with magnetic beads;

(3) Libraries were assayed for concentration using a Qubit and fragment size using an Agilent 2100.

Table 8. Library QC results

| **No.** | **Sample name** | **Index No.** | **Concentration (ng/μL)** | **Main peak length (bp)** |
| --- | --- | --- | --- | --- |
| 1 | DM | E12 | 16.1 | 430 |
| 2 | NM | D12 | 5.58 | 426 |
| 3 | NMI | SI-TT-G8 | 19.7 | 429 |
| 4 | DMI | SI-TT-H9 | 33.2 | 463 |

The Agilent 2100 Bioanalyzer detection peaks are shown below:

Figure 9. Quality control analysis of DM group mouse library

Figure 10. quality control analysis of the NM group mouse library

Figure 11. Quality control analysis of the NMI group mouse library

Figure 12. Quality control analysis of the DMI group mouse library

From Table 8 and Figures (9-12), it was found that the concentrations and major peaks among the samples of the library constructs met the quality control requirements and could be continued for Illumina sequencing.

2.2.3 On machine sequencing

Complete cluster generation and first direction sequencing primer hybridization according to the Illumina User Guide, and put the flow cell carrying the cluster on the machine. Select the paired end program for dual end sequencing. The sequencing process is controlled by Illumina's data collection software and real-time data analysis is performed.

3 Statistical methods

Using Cell Ranger to use barcode and MUI to count the number of cells in each sample, as well as the number of cell reads and detected genes; Cell clustering and gene analysis were conducted using the commonly used scRNA seq analysis process and "R" language provided by the Chromium system platform of 10X Genomics. Gene difference analysis was performed using | logFC |>0.25; Perform statistical analysis with Pct.1>0.25.

**Figure 13. all_top_marker_heatmap**

**Table 9. Marker genes for cell annotation**

| **Cluster** | **Maker genes** | | | **Cell type** |
| --- | --- | --- | --- | --- |
| 0 | ttn | ryr2 | trdn | Cardiomyocytes |
| 1 | CDH5 | Pecam1 | adgrf5 | endothelial cells |
| 2 | dcn | pdgfra | col1a2 | fibroblasts |
| 3 | ttn | ryr2 | trdn | Cardiomyocytes |
| 4 | ttn | ryr2 | trdn | Cardiomyocytes |
| 5 | CDH5 | Pecam1 | adgrf5 | endothelial cells |
| 6 | ttn | ryr2 | trdn | ventricular cardiomyocytes |
| 7 | Cspg4 | Pdgfrb | Vtn | pericytal cells |
| 8 | CSF1R | CD163 | C1QA | Cardiac glial cells |
| 9 | CDH5 | Pecam1 | adgrf5 | endothelial cells |
| 10 | CDH5 | Pecam1 | adgrf5 | endothelial cells |
| 11 | ttn | ryr2 | trdn | Cardiomyocytes |
| 12 | dcn | pdgfra | col1a2 | fibroblasts |
| 13 | CSF1R | CD163 | C1QA | Cardiac glial cells |
| 14 | CDH5 | Pecam1 | adgrf5 | endothelial cells |
| 15 | LMOD1 | MYH11 | ACTA2 | smooth muscle cells |
| 16 | dcn | pdgfra | col1a2 | fibroblasts |
| 17 | msln | bnc1 | lrrn4 | epicardial cells |
| 18 | CDH5 | Pecam1 | adgrf5 | endothelial cells |
| 19 | msln | bnc1 | lrrn4 | epicardial cells |
| 20 | cxcr2 | csf3r | cd177 | neutrophils |
| 21 | gfra3 | plp1 | kcna1 | glial cells |
| 22 | CD19 | CD79A | PAX5 | B lymphocytes |
| 23 | Cd3e | Cd3d | Lef1 | T cells |
| 24 | Cspg4 | Pdgfrb | Vtn | pericytal cells |
| 25 | Plp1 | Kcna1 | Kcna1 | Cardiac glial cells |
