# Supplementary material for: Txnip/PDK4‐Mediated Hyperdependence on Fatty‐Acid Oxidation Amplifies Susceptibility of Diabetic Myocardium to Ischemia–Reperfusion Damage
Source: J Diabetes. 2026 Oct 20;18(10):e70277. doi: 10.1111/1753-0407.70277 (PMC13681344; doi:10.1111/1753-0407.70277)
Supplement: Supplementary file 6 [file JDB-18-e70277-s007.docx]

*Appendix 4.*

**Single cell result analysis for confirming the localization of C6 subgroup tissue**

The common Marker genes of myocardial cells were obtained through the CellMarker website. Through these genes, 26 cell subpopulations can be divided into myocardial cell subpopulations and non myocardial cell subpopulations. We further selected TTN as our primary marker for myocardial cells based on the expression level of the Marker gene.

Figure 1. Expression of Marker gene in myocardial cells in 26 cell subpopulations

In order to better identify C6 from the expression of genes, the top ten genes with the highest expression level in the C6 subgroup were selected to study their expression among different subgroups. Although the expression levels of Myh6 and GM42418 are higher, their differences among different subgroups are not as significant as Myl2, Myl3, and Mb; Due to Mb (myoglobin) being a non-specific antigen, it is not suitable as the first choice for locating cell subpopulations.

Figure 2. Expression of C6 highly expressed genes in 26 cell subpopulations

Due to the need to locate the location of C6 cell subpopulations from myocardial cells, the Marker genes of C6 subpopulations directly obtained through single cell sequencing should also have significant differences among the five myocardial cell subpopulations. Xirp2, as a Marker gene in the C6 subgroup, has a higher expression level in the C6 subgroup compared to other cell subgroups.

Figure 3. Marker genes in different cell subpopulations

Both Ttn+Myl3+Xirp2 and Ttn+Myl2+Xirp2 can effectively screen out the C6 subgroup in the cell subpopulations. Compared to Ttn+Myl2+Xirp2, Ttn+Myl3+Xirp2 can better classify cell subpopulations into C6 subpopulations, myocardial cell subpopulations excluding C6, and non myocardial cell subpopulations.

Figure 4. Expression of Ttn, Myl3, and Xirp2 in 26 cell subpopulations

Figure 5. Expression of Ttn, Myl2, and Xirp2 in 26 cell subpopulations

In summary, we used Ttn+Myl3+Xirp2 as a marker gene for spatial localization of C6 cell subpopulations.
