# Supplementary material for: Txnip/PDK4‐Mediated Hyperdependence on Fatty‐Acid Oxidation Amplifies Susceptibility of Diabetic Myocardium to Ischemia–Reperfusion Damage
Source: J Diabetes. 2026 Oct 20;18(10):e70277. doi: 10.1111/1753-0407.70277 (PMC13681344; doi:10.1111/1753-0407.70277)
Supplement: Supplementary file 7 [file JDB-18-e70277-s003.doc]

*Appendix 5.*

**Experimental report of SweAMI fluorescence in situ hybridisation triple staining (FISH) of paraffin sections**

**1.** **Experimental equipment and reagents**

**1.1.** **Experimental equipment**

| **designation** | **factory** | **models** |
| --- | --- | --- |
| Dehydrator | DIAPATH | Donatello |
| Glass slide | Servicebio |  |
| Cover glass  Pathological microtome | Jiangsu Shitai experimental equipment Co., LTD  Shanghai Laika Instrument Co., LTD | 10212432C  RM2016 |
| Enzyme-free centrifuge tube | Servicebio | EP-150-M |
| Enzyme-free centrifuge tube | Servicebio | TSY-B |
| Enzyme-free centrifuge tube | Servicebio | MV-100 |
| Pipette gun | Dragon | KE0003087/KA0056573 |
| Immunohistochemistry pen | Servicebio | G6100 |
| Refrigerator | Qingdao Haier Co., LTD | BCD-192TGN |
| Orthostatic Fluorescence Microscope | Nikon | NIKON ECLIPSE CI |
| Imaging system  Thermostat  Autoclave | Nikon  labotery  Panasonic Health Medical | Nikon DS-U3  GSP-70  MLS-3751L-PC |
| Embedding machine  Frozen table  Tissue machine | Wuhan Junjie Electronics Co., LTD  Wuhan Junjie Electronics Co., LTD  Zhejiang Jinhua Kedi Instrument Equi pment Co., LTD | JB-P5  JB-L5  KD-P |

**1.2.** **Principal experimental reagent**

| **Reagent** | **Manufacturer's** | **article number** | | **dilution ratio** |
| --- | --- | --- | --- | --- |
| DEPC  4% paraformaldehyde (DEPC water)  paraffin | Amresco  Servicebio  Sakura | | E174  G1113 |  |
| Anhydrous ethanol | Sinopharm Group Chemical reagent Co., LTD | | 100092683 |  |
| Environmentally friendly dewaxing transparent liquid | Servicebio | | G1128 |  |
| PBS  20×SSC eluent | Servicebio  Servicebio | | G0002  G3016-4 |  |
| BSA | Servicebio | | G5001 |  |
| Protease K | Servicebio | | G1234 |  |
| DAPI | Servicebio | | G1012 |  |
| Anti-fluorescence Quenching sealing tablets | Servicebio | | G1401 |  |
| Hybridization buffer | Servicebio | | G3016-3 |  |

### 1.3 Probe information and repair conditions

| Repair condition | Digestive condition | Probe name | Probe sequence (5 '-3') | Probe concentration | Hybridization temperature | Name of the corresponding branch probe | Name of the corresponding signal probe |
| --- | --- | --- | --- | --- | --- | --- | --- |
| Medium fire 7 minutes, cease fire 7 minutes, medium low fire 6 minutes | 15 minutes | TTN | CGGTTAATCACAAGTCGCTGTTTGG, TCAGGGTATGCTTCGGCAATCAGTAA, GGCTTCATAGTATCCCTCTTCCGTCC, CCAAGGGCAGGAATAGTGAAGGAGTA, TGGTGGTCTGAGTCTTGCGAATGAG | 500nM | 40 | Branch probe 1 | CY3 signal probe 1 |
|  |  | XIRP | TCAATGACAATCTTTACAGTGCCCTTAG, TTCTAAGGCTTTTCTCAAGAGCATCATT, TCTTTGCTCTCAGTCCAGATACCCATT, CTTTATTAGAATACTGCTTTTCCTGACTGA, AGAACTGTTGTAAAAATGCTTCCCTGTG | 500nM | 40 | Branch probe 2 | FITC signal probe 2 |
|  |  | MYL3 | CTTTGGCATCATCCTTCTTGGGCT, CGTATGTGATCTTCATCTCGCCCTTG, AGCACCTCTGCCTGGGTAGGATTCT | 500nM | 40 | Branch probe 4 | CY5 signal probe 4 |

(Note: The order of adding the probe sample is in accordance with the order of writing the probe)

**2. Procedures of SWEAMI-fluorescence in situ hybridization experiment in** **paraffin sections**

2.1 Tissue fixation: After the tissue is removed and washed, it is immediately placed in the in situ hybridization fixation solution and fixed at 4° for more than 12h.

2.2 Dehydration embedding: After the tissue fixation is completed, it is dehydrated with gradient alcohol, then dipped in wax and embedded.

2.3 Paraffin slicing: sliced by microtome, slices spreaded by machine, place slices in the oven at 60°C for 1 h.

2.4 Paraffin sections dewaxed and dehydrated: the sections are sequently immersed in environmentally friendly dewaxing transparent liquid Ⅰ 15min- Environmentally friendly dewaxing transparent liquid Ⅱ 15min- anhydrous ethanol Ⅰ 5min- anhydrous ethanol Ⅱ 5min-85% alcohol 5min-75% alcohol for 5min and DEPC water.

2.5 Digestion: According to the length of tissue fixation time, the section is repaired in the repair solution, the repair conditions are shown in the table above, and the natural cooling. After the pen circle is organized, protease K(20ug/ml) is added to digestion at 37° according to different indexes of different tissues. The digestion time is shown in the table above. After rinsing with pure water, wash with PBS for 3 times × 5min.

2.6 Pre-hybridization: Add pre-hybridization solution and incubate at 37°C for 1h.

2.7 Hybridization: Pour out the pre-hybridization solution, drop the hybridization solution containing the probe, and hybridization overnight in the incubator. Hybridization conditions are shown in the table above.

2.8 Washing after hybridization: wash the hybridization solution, 2×SSC, 37°C for 10min, 1×SSC, 37°C for 2×5min, 0.5×SSC at room temperature for 10min. If there are more non-specific hybrids, formamide can be added for washing.

2.9 Drop the corresponding branch probe hybridization: gently dry the slice, drop the preheated corresponding branch probe hybridization solution (see the table above and attached table) (60μL), and horizontally place in a wet box at 40℃ for hybridization for 45 min. During this process, 50ml 2 × SSC is added to the bottom of the wet box to prevent drying.

2.10 Washing after hybridization: Pour the hybridization solution, and rinse the slices successively with 2× SSC, 1× SSC, 0.5× SSC, 0.1× SSC preheated at 40°C for 5 min. Note: If there are more non-specific hybrids, you can increase the washing time, frequency and adjust the formamide concentration in the hybrid solution in this step.

2.11 Drop the corresponding signal probe: Drop the hybrid solution containing the signal probe (see the table above and the attached table), and incubate at a dilution ratio of 1:400 ，42 ° for 3h. After washing, it is washed by the following SSC in sequence: 2×SSC, 37°C for 10min, 1×SSC, 37°C for 2×5min, 0.5×SSC 37° for 10min.

2.12 Hybridization: Pour out the pre-hybridization solution, drop the hybridization solution containing the probe, and hybridize overnight in the incubator. Hybridization conditions are shown in the table above.

2.13 Washing after hybridization: wash the hybridization solution, 2×SSC, 37°C for 10min, 1×SSC, 37°C for 2×5min, 0.5×SSC at room temperature for 10min. If there are more non-specific hybrids, formamide can be added for washing.

2.9 Drop the corresponding branch probe hybridization: gently dry the slice, drop the preheated corresponding branch probe hybridization solution (see the table above and attached table) (60μL), and horizontally place in a wet box at 40℃ for hybridization for 45 min. During this process, 50ml 2 x SSC is added to the bottom of the wet box to prevent drying.

2.14 Washing after hybridization: Pour the hybridization solution, and rinse the slices successively with 2× SSC, 1× SSC, 0.5× SSC, 0.1× SSC preheated at 40°C for 5 min. Note: If there are more non-specific hybrids, you can increase the washing time, frequency and adjust the formamide concentration in the hybrid solution in this step.

2.15 Drop the corresponding signal probe: Drop the hybrid solution containing the signal probe (see the table above and the attached table), and incubate at a dilution ratio of 1:400，42 ° for 3h. After washing, it is washed by the following SSC in sequence: 2×SSC, 37°C for 10min, 1×SSC, 37°C for 2×5min, 0.5×SSC 37° for 10min.

2.16 Hybridization: Pour out the pre-hybridization solution, drop the hybridization solution containing the probe, and hybridize overnight in the incubator. Hybridization conditions are shown in the table above.

2.17 Washing after hybridization: wash the hybridization solution, 2×SSC, 37°C for 10min, 1×SSC, 37°C for 2×5min, 0.5×SSC at room temperature for 10min. If there are more non-specific hybrids, formamide can be added for washing.

2.18 Drop the corresponding branch probe hybridization: gently dry the section, drop the preheated corresponding branch probe hybridization solution (see the table above and attached table) (60μL), and horizontally place in a wet box at 40℃ for hybridization for 45 min. During this process, 50ml 2 x SSC is added to the bottom of the wet box to prevent drying.

2.19 Washing after hybridization: Pour the hybridization solution, and rinse the slices successively with 2× SSC, 1× SSC, 0.5× SSC, 0.1× SSC preheated at 40°C for 5 min. Note: If there are more non-specific hybrids, you can increase the washing time, frequency and adjust the formamide concentration in the hybrid solution in this step.

2.20 Drop the corresponding signal probe: Drop the hybrid solution containing the signal probe (see the table above and the attached table), and incubate at a dilution ratio of 1:400.42 ° for 3h. After washing, it is washed by the following SSC in sequence: 2×SSC, 37°C for 10min, 1×SSC, 37°C for 2×5min, 0.5×SSC 37° for 10min.

2.21.DAPI restaining core: drop DAPI dye solution into the section, incubate in dark for 8min, rinse and add anti-fluorescence quenching sealing tablet.

2.22. Photographic examination: Sections are observed and images are collected under Nikon's standing fluorescence microscope. (UV excitation wavelength 330-380nm, emission wavelength 420nm, emitting blue light; FAM(488) emits green light with excitation wavelength 465-495nm and emission wavelength 515-555 nm. CY3 red light excitation wavelength of 510-560, emission wavelength of 590nm, red light. CY5 powder light excitation wavelength 646nm, emission wavelength 664nm, powder light)

**3. Technical principles**

Using SweAMI technology, specific oligonucleotides are tail-treated as probes. By using specific branch probes as intermediate hybridization medium, more fluorescence signal probes can be combined to form more fluorescent labels. Localization and quantification of target gene-probe hybrids are realized.

**4. Interpretation of the results of in situ hybridization with SweAMI probe in paraffin sections**

The nuclei stained by DAPI are blue under ultraviolet excitation, and the positive expression is the corresponding fluorescein labeled fluorescence. FAM(488) is green light, cy3 is red light and CY5 is pink light. The intensity of fluorescence varies according to the expression quantity.

**Note: All the reagents and instruments mentioned above need to be used in the Rnase free environment after DEPC treatment in the RNA in situ hybridization experiment.**
